# The Effects of Resigning GPs on Patient Healthcare Utilization and Some Implications for Health

**DOI:** 10.1002/hec.4941

**Published:** 2025-01-30

**Authors:** Daniel Monsees, Matthias Westphal

**Affiliations:** ^1^ RWI Essen Essen Germany; ^2^ Leibniz Science Campus Ruhr Essen Germany; ^3^ Faculty of Business Administration and Economics, Chair for Economic Policy University in Hagen Hagen Germany

**Keywords:** general practitioners, healthcare expenditure, healthcare utilization, primary care

## Abstract

We study the effects of general practitioners' (GPs') resignations on their patients' healthcare utilization and diagnoses in an event‐study setting. Using claims data from a large German statutory health insurance, we find that after physicians leave, their former patients persistently reduce their primary care utilization, only partially substituting it with specialist visits and hospital care. Because patients find a new GP already 1.1 quarters after the old resigns, on average, the persistent effects must be explained through the new GP. Indeed, the new GP serves more patients but performs less diagnostic testing. Our results reveal a substantial decrease in diagnoses of many relevant chronic conditions (such as congestive heart failure and diabetes), suggesting that disruptions may have adverse consequences for the efficiency of the healthcare system. This indicates that continuity in primary care is pivotal and shows that the GP has an essential role in healthcare delivery, particularly in healthcare systems such as Germany, where GPs often have a high workload and little consultation time.

## 1 Introduction

A close and long‐lasting relationship between general practitioners (GPs)^1^ and their patients is generally regarded as desirable and an essential factor in providing high‐quality ambulatory care (Baker et al. 2020; Nyweide et al. 2013; Schuettig and Sundmacher 2022; Saultz and Lochner 2005). Patients may develop trust in their GPs over time while GPs gather specific and potentially important knowledge about their patients' underlying health—allowing them to make informed decisions about the best treatment options. The current demographic shift toward older populations in Western countries also affects the healthcare sector workforce. While in 2012, 9% of the practicing GPs in Germany were above the age of 65, this share rose to 20% in 2022 (Bundesärztekammer 2023). These older physicians will soon resign, challenging the continuity of care for patients. This is particularly problematic in the German setting where no substitute for GPs services in primary care—like nurse practitioners—exists. However, if the new GPs have superior knowledge and treatment styles compared to their predecessors, changing GPs may benefit patients. Whether this is the case also depends on how quickly patients search for new doctors and how accessible primary care is in the healthcare system. Therefore, which forces prevail in the particular setting is an important empirical question we study in this paper.

Specifically, we evaluate the effects of a disruption of the patient‐provider relationship on healthcare utilization, changes in the practice styles between the old and new GP, and mortality using detailed administrative claims data from a German statutory health insurance comprising almost 9 million insurees. We assess the health and healthcare utilization consequences for patients whose primary care provider resigns from their profession (e.g., due to retirement) in an event‐study setting, where we center calendar time on the exiting period of the leaving GP.

Similar approaches have been used to evaluate the effects of disrupting the patient‐provider relationship in various healthcare systems. Most evidence comes from the US using either data on Medicare recipients aged 65 and older (for instance, Fadlon and van Parys, 2020; Kwok 2019; Sabety, Jena, and Barnett 2021; Zhang 2022) or individuals in Medicaid who cannot afford regular health insurance (Staiger 2022). Evidence outside the US is more scarce, comprising of Bischof and Kaiser (2021), Simonsen et al. (2021), and Zocher (2024), who study Switzerland, Denmark, and Austria, respectively. For the US, the literature documents significant reductions in primary care visits after a GP's exit. This reduction goes along with an increase in specialist visits. Since both these effects are persistent, this suggests a shift in healthcare utilization toward specialists. An increase in emergency department visits or hospitalizations is only found in the short term. For Switzerland, Bischof and Kaiser (2021) report similar effects. They find a persistent decrease in GP visits and an increase in specialist visits. Although hospital visits increase, mortality does not seem affected. Likewise, results for Austria suggest a significant increase in healthcare spending for inpatient and outpatient services of affected patients (Zocher 2024). In Denmark, a higher level of regulation might explain why the transition between physicians is much smoother (Simonsen et al. 2021). They find GP visits decline at the expense of increased hospitalizations for chronic conditions. The latter arguably results from reassessing patients' health by the absorbing GP or primary care provider, potentially benefiting the patients.

We contribute to this recent literature by analyzing an interesting healthcare setting that may complement existing evidence, especially in showing that disruptions can have adverse consequences for healthcare accessibility that may even affect health in the long run. We study GP exits within German social health insurance. Notably, three of its features distinguish our setting from the literature and may thereby explain this paper's pronounced and partially novel effects. First, the insurance plans of the social health insurance that we study do not exhibit any deductibles or copayments as is the case, for example, in Bischof and Kaiser (2021). Deductibles can cause confounding effects as they censor healthcare utilization from below. Second, although we may not cover the disproportional high earners and civil servants from private health insurance, we otherwise study a general population that is, almost unrestricted regarding age, employment, and socioeconomic characteristics. Finally, although Germany has one of the highest physician densities, rendering the healthcare system highly accessible in principle (Blümel et al. 2020), GPs generally tend to have a high workload due to a large healthcare demand (OECD 2023), with one of the lowest consultation time per patient compared to other countries (Deveugele et al. 2002; von dem Knesebeck et al. 2019) resulting in comparatively higher work‐related stress levels of German GPs (Siegrist et al. 2010; Voltmer et al. 2024).

Our findings are compatible particularly with the latter feature and suggest a significant and persistent reduction in the utilization of GP services, mainly driven by having regular contact with the GP (where we find a 5% reduction in the probability of visiting the GP in a given quarter). While we find evidence for substituting GP with specialist services, hospital services, particularly adverse emergency hospitalizations, seem to be a more critical substitute in the short run. In contrast, the persistence of preventable hospitalizations (that can be avoided by good ambulatory care) indicates an increased inefficiency in healthcare provision. As affected patients find a new GP 1.1 quarters after the exit of the former one, our persistent effects must be explained through the (relationship with the) new GPs and the frequency of primary care checkups. We find that new GPs serve more patients (mechanically reducing the average potential consulting time per patient) and are more likely part of a group practice. They also perform less diagnostic testing (regarding blood counts and protein tests) and prescribe more preventive drugs against cardiovascular diseases (ACE inhibitors) as a potential consequence of reduced time. Generally, practice style differences between old and new physicians cannot explain these effects, suggesting that new patients are treated differently than the new GP's average patient. Although we do not find higher patient mortality rates, we find evidence that GP disruption reduces healthcare quality. Following the old GP's exit, the new one detects fewer chronic diseases (such as congestive heart failure and diabetes). Most likely, these missed diagnoses directly result from fewer primary care checkups. Documenting these missed diagnoses is a novel finding in the literature and is compatible with increased work‐related stress of younger GPs and for consultations with new patients (von dem Knesebeck et al. 2019).

Our paper proceeds as follows. In Section 2, we describe the institutional details of the German healthcare setting before we present the employed administrative data in Section 3. Section 4 details our event‐study regression model and the necessary assumptions. We document our detailed results in Section 5. Finally, Section 6 concludes.

## 2 Institutional Setting

Health insurance is compulsory in Germany. This means that citizens have to be enrolled either in statutory health insurance (SHI; about 87% of the population) or—if they meet certain criteria that relate to earned income and employment—in private health insurance (PHI; about 10.8%, see Blümel et al. 2020).^2^ While privately insured individuals have to pay for services upfront, which will be reimbursed by their insurance afterward, individuals in the SHI encounter almost no (out‐of‐pocket) fees for physician services. Individuals covered by the SHI may receive treatment from any physician contracted with the SHI (which is the case for more than 66% of the physicians). This liberal principle applies to general and specialist physicians—a mandatory gatekeeping function does not exist. Together with the high physician density, this results in a high accessibility of healthcare services (Blümel et al. 2020), which should make it relatively easy for patients to switch from one physician to another and open the possibility of substituting GP with specialist services.

Germany has above‐average outpatient contacts within the EU, with about 9.9 contacts per capita in 2018 (Blümel et al. 2020). Despite the high theoretical accessibility, the high contacts cause GPs to have a high workload, which is reflected in high waiting times (Werbeck, Wübker, and Ziebarth 2021), and a GP workforce that scores higher on work‐related stress than GPs in other countries (Siegrist et al. 2010; Voltmer et al. 2024). German GPs primarily work in solo practices (with 53% of GPs working in single practices in 2023 (Kassenärztliche Bundesvereinigung (KBV), 2024)), albeit there is an increasing tendency for employment in group practices, especially medical care centers. This tendency also explains the decreasing total number of general practices (from 32,319 in 2012 to 26,175 in 2022, Kassenärztliche Bundesvereinigung (KBV), 2022), while the total number of GPs is relatively stable (increasing from 54,172 in 2012 to 55,050 in 2022 Kassenärztliche Bundesvereinigung (KBV), 2024). Still, the dominance of solo practices means that the exit of individual physicians likely results in the closure of practice and, thus, a significant disruption in the continuity of care for patients. Concerning the exits themselves, there is little to no regulation. GPs can revoke their license to practice until the end of each quarter. There are no regulations concerning when GPs have to inform their patients.^3^ In general, GPs are required to provide medical records to patients or other physicians if they agree to it (Kamps 2010). This also includes retired physicians, who must store medical records for 10 years. From anecdotal evidence, most GPs ask new patients to sign a form allowing the GP to demand the medical records from the old GP.

## 3 Data

We use administrative claims data from a large statutory health insurance covering about 10.6% of the German population (Grobe, Braun, and Szecsenyi 2022), with coverage rates that vary between 5.5% and 17.4% across the federal states (Augurzky et al. 2022). Although the data does not necessarily represent the German population, we cover nearly all contracted German physicians (GPs and specialists) registered in Germany. The data contains extensive information on the healthcare services patients use, including outpatient and in‐patient services, ambulatory and stationary hospital care. Importantly, we can identify the individual providers for each healthcare service. These are important features compared to other administrative health insurance data in Germany, which allow us to draw a comprehensive picture of the effects across all areas of healthcare. We use quarter‐level data from the years 2010–2019.

### 3.1 Identifying General Practitioners

**FIGURE A1:**
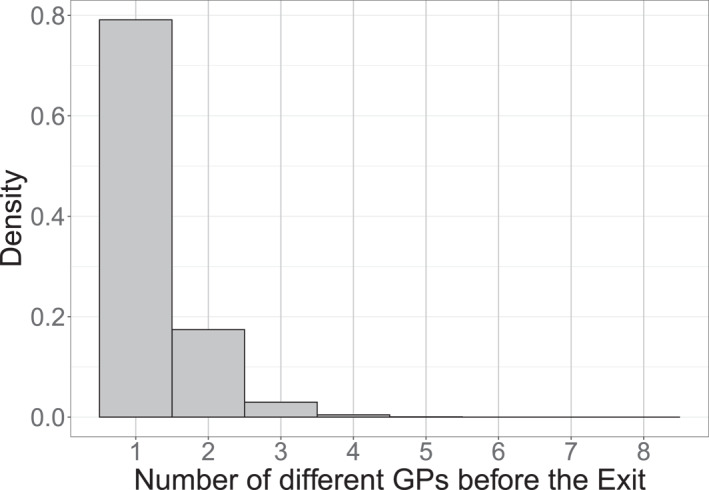
Number of different GPs before the exit per Patient. 383,502 observations of 383,502 patients. Mean = 1.25.

The data allows us to identify physicians, their practices, and their specializations. Although we do not directly observe when physicians close their practice, we can identify the last quarter in which a physician bills any service to the health insurance.^4^ We define this as the quarter when the physician discontinues their service. We are mainly interested in general practitioners (GPs) exiting from the first quarter of 2012 to the last quarter of 2016. We refer to this group of GPs henceforth as *leaving physicians*, of whom we identify 7376 in our dataset. Because patients are not bound to any provider in Germany, we do not observe each individual's leading provider directly. However, we define the patients' leading provider as the GP who provides the most healthcare services (billing the most fee schedule positions^5^) to patients for at least four consecutive quarters. Given that patients are free to visit any GP in Germany, the idea of a *leading provider* might not be realistic, however, as Figure A1 (depicting the number of GPs the patients see before the exit) shows, almost 80% of patients see only one GP before the exit and patients seeing more than 3 GPs before the exit are an exception.

### 3.2 Sample

We only include patients exposed to exactly one GP exit in the study period (between 2010 and 2019) if that exit occurred between 2012 and 2016. Hence, we drop individuals who experienced no GP exit or multiple exits between 2012 and 2016. We also exclude individuals whose GP exited before 2012 or after 2016. Additionally, we restrict the sample to patients who were continuously insured during the whole study period and are aged between 18 and 80 years at the beginning of the study period.^6^ This leaves us with 15,340,080 patient quarter observations of 383,502 individuals.

### 3.3 Outcomes

Table 1 provides an overview of our main estimation sample and contrasts their characteristics with a sample of individuals who do not experience any GP exit in the study period. For both samples we compare values from the first quarter in 2010, leaving us with 383,502 observations in the estimation sample and 4,940,169 observations of individuals whose GP does not exit.

**TABLE 1:** Descriptive statistics: Exit sample versus non‐exit sample.

	Estimation sample (exposed to a leaving GP) 6 quarters before exit		Non‐exit sample (not exposed to a leaving GP, dropped in the analyses)		SMD
	Mean	SD	Mean	SD	
Patient characteristics					
Birth year	1958.026	15.641	1959.828	15.730	−0.081
Age	51.974	15.641	50.128	15.693	0.083
Female	0.602	0.490	0.592	0.492	0.014
Rural	0.328	0.469	0.338	0.473	−0.016
Healthcare utilization					
Number of GP visits	1.640	2.190	1.364	2.133	0.090
Any GP visit	0.677	0.468	0.549	0.498	0.187
GP costs (€)	44.500	66.230	37.295	59.311	0.081
Number of specialist visits	1.567	2.862	1.310	2.666	0.066
Any specialist visit	0.486	0.500	0.406	0.491	0.113
Specialist costs (€)	64.763	204.927	54.041	184.057	0.039
Any hospital visit	0.028	0.165	0.024	0.154	0.017
Any emergency hospital visit	0.014	0.116	0.012	0.106	0.014
Any ambulatory care sensitive condition	0.002	0.050	0.002	0.047	0.004
Diagnoses					
Myocardial infarction	0.008	0.088	0.006	0.080	0.005
Congestive heart failure	0.020	0.142	0.017	0.128	0.009
Peripheral vascular disease	0.028	0.165	0.024	0.152	0.010
Cerebrovascular disease	0.029	0.167	0.024	0.154	0.011
Dementia	0.003	0.057	0.003	0.051	0.003
Chronic pulmonary disease	0.102	0.303	0.085	0.279	0.028
Rheumatoid disease	0.019	0.137	0.015	0.120	0.012
Peptic ulcer disease	0.006	0.076	0.004	0.065	0.005
Mild liver disease	0.051	0.220	0.042	0.201	0.017
Diabetes without complications	0.065	0.247	0.053	0.224	0.023
Diabetes with complications	0.017	0.130	0.016	0.127	0.002
Hemiplegia or paraplegia	0.006	0.079	0.005	0.071	0.004
Renal disease	0.015	0.120	0.012	0.109	0.008
Cancer (any malignancy)	0.039	0.193	0.030	0.171	0.018
Moderate or severe liver disease	0.001	0.025	0.001	0.025	0.0003
Cancer (metastatic solid tumor)	0.002	0.049	0.002	0.043	0.003
AIDS	0.001	0.024	0.001	0.027	−0.001
Tests and prescriptions					
Any blood count	0.013	0.113	0.011	0.106	0.010
Any total protein	0.004	0.066	0.004	0.065	0.002
Any beta blocker	0.090	0.287	0.069	0.254	0.056
Any ACE inhibitor	0.068	0.251	0.055	0.229	0.036
Any antibiotics	0.013	0.112	0.010	0.100	0.016
Any sick note	0.148	0.356	0.129	0.336	0.039
Observations	383,502		4,940,169		

*Note:* The estimation sample consists of individuals who are continuously insured and who all experience a GP exit between 2012 and 2016. The untreated sample consists of individuals who are continuously insured and who experience no GP exit between 2010 and 2019. For both samples observations from the first quarter in 2010 are presented. *Rural* indicates individuals living in a county with less than 75% of the municipalities having a population density of more than 150 inhabitants per $km^{2}$ (BBSR 2023). Diagnoses indicate whether an individual has ever received the given diagnosis, based on the Charlson Comorbidity Index (Charlson et al. 1987). Complete Blood Count as defined by EBM No. 32122. Total Protein as defined by EBM No. 32056. ACE Inhibitors include all prescriptions with ATC C09a and C09b. Beta Blockers include all prescriptions with ATC C07. Antibiotics include all prescriptions with ATC J01. SMD refers to the standardized mean differences and is calculated as follows: $SMD=\frac{Mean_{1}\;-\;Mean_{0}}{\sqrt{SD_{1}\;+\;SD_{0}}}$ (where the index 1 indicates the respective statistic for the estimation sample, while 0 refers to the untreated sample).

We base our analysis on the estimation sample and drop patients whose GP never quits. This is not only due to computational convenience but also because the observed and unobserved patient characteristics between exiting and remaining GPs may differ. This would be especially problematic if patients whose GP never quits are different in trends from patients whose GP does exit, as it would be a violation of the common trend assumption. This thread needs to be taken seriously as leaving physicians are most likely older than GPs who do not leave and serve older patients with higher health care needs. While we have extensive medical information on insured individuals, the socioeconomic information is limited. The average individual in the estimation sample is born in 1958, translating to an age of 52 Years in 2010, 60% of the sample is female, and one‐third of individuals live in rural counties. We use a representative 2010 sample of the SOEP survey data, applying only the age restriction. We find respondents are 50% female, 31% live in rural areas, and are, on average, born in 1960 (with an average age of 50) (Goebel et al. 2023).^7^ Individuals in the SOEP are also asked if they have visited a GP in the previous 3 months, which is the case for 70.5% of respondents in our sample (compared to 67.7 in the estimation sample). Although the data are certainly not representative in all (unobserved) dimensions, the few observed dimensions suggest, if anything, that the sample is not too different from the general population.

The main outcome variable is an indicator equal to one if an individual ever visited any GP during a quarter. In our estimation sample, 67% of patients see a GP on average before the exit. Similarly, we define an indicator for specialist services, with specialists being any physician who is not a GP, gynecologist, or pathologist, occurring for 48% of patients. *Emergency Visits* comprise all hospital visits that occurred without a referral from a physician, again as an indicator on the quarter level, with an average of 1.4% of patients. Other forms of hospital visits occur for 3% of patients. Likewise, 0.2% of individuals in the estimation sample are hospitalized with an ambulatory care‐sensitive condition—as defined by Albrecht and Sander (2015)—in a given quarter. Next to this, we include costs for physician services—both GPs and specialists—per patient per quarter. The amount of money paid to a physician for treating a certain patient is based on a complex calculation involving multiple actors in the German healthcare system. Since it is impossible to calculate the exact amount with the data at hand, we calculate a proxy for the health insurance expenses based on the services provided by the physician, which we argue is sufficiently close. As a result, we observe average quarterly costs of €44.5 per patient for GP visits and €64.7 for specialist visits. We also include the number of ambulatory care visits per quarter for GPs and specialists, with an average of 1.64 and 1.56, respectively.

To assess proxies of healthcare quality that could be affected by the transition from leaving to absorbing GP, we consider diagnoses based on the Charlson Comorbidity Index (Charlson et al. 1987). Here, we present an indicator if the respective diagnosis was documented in the given quarter, irrespective of whether it was diagnosed for the first time. Lastly, we include tests and prescriptions in our analysis. These should reveal how physicians diagnose diseases and how they decide to treat them. As before, the outcome variables are coded as one in the quarters where a given individual's respective outcome is observed. Blood counts contain various measures supporting the diagnosis of different diseases and are performed for 1.3% of individuals in a given quarter. Total protein is tested for 0.4% of patients in a given quarter and is part of the diagnosis of heart failure. For prescriptions, we focus on those related to heart conditions, as these are the most common in our sample (see above). Beta Blockers and ACE Inhibitors are used for treating high blood pressure, that is, one of the earliest risk factors of cardiovascular disease (Strauss, Hall, and Narkiewicz 2023) with evidence that especially ACE Inhibitors are underused (Brooks et al. 2018). More than 9% of individuals receive a prescription for Beta Blockers, 6.8% receive prescriptions for ACE Inhibitors. One percent of patients receive antibiotics in a given quarter, a class of drugs generally thought of as being prescribed too often. Lastly, 14.8% of patients receive a sick note.

Mean differences between the estimation and the non‐exit sample are not negligible. For instance, the likelihood of visiting a GP is 13 percentage points higher in a given quarter. The remaining variables also indicate worse health outcomes of the estimation sample. However, as retirement is probably the leading cause for the GPs to resign, GPs in the estimation sample are likely much older than those in the comparison group. This age gap also translates to patients who are almost 2 years older. To statistically assess these differences, we provide standardized mean differences (SMD) in the last column of Table 1. Almost all differences are below 10% of a standard derivation, and most are below 5%. Meaningful differences are only evident for outpatient care utilization; our estimation sample is more likely to visit GPs and specialists. Also, our estimation sample is 2 years older than the non‐exit sample. While this indicates that one needs to be cautious when transferring our results to a general population, this also shows that leaving GPs and their patients are somewhat different.

## 4 Empirical Strategy

To estimate the potential repercussions on patients that could be caused by leaving GPs, we apply a standard event study model of the following form:

(1) $$Y_{it}=\alpha_{i}+\lambda_{t}+\beta^{\mathrm{BIN}}\sum_{j\leq -9}e\left(j\right)+\sum_{\begin{array}{c} j\geq -8;\\ j\neq -6 \end{array}}\beta_{j}^{ES}e\left(j\right)+\epsilon_{it},$$

where $Y_{it}$ is the respective outcome (such as measures on healthcare utilization) of individual i in quarter t. We regress this outcome on $\alpha_{i}$ and $\lambda_{t}$, which are individual‐specific and quarter effects, respectively. Additionally, we include indicators for the relative time since the exit of the original GP as our regressors of interest. We denote these regressors by $e\left(j\right)≔1\left[t-q_{i}=j\right]$. As before, t is the usual calendar time, while $q_{i}$ denotes the quarter of exit of the GP of patient i. The coefficients $\beta_{j}^{ES}$ are the parameters of interest and capture the differences in the outcomes for the $j^{th}$ event time with respect to quarter −6, that is, 1.5 years before the GP stops practicing. As is convenient in the literature, we bin the lowest event times (before −8) together (i.e., we include $\sum_{j\leq -9}e\left(j\right)$ as the sum of mutually exclusive dummies as a further dummy) but leave the highest ones (up to 31 quarters after the GP's exit) unrestricted. We only discuss event‐time coefficients from −8 to 12, where our panel is balanced (as we only consider GP exits from 2012 to 2016). Finally, $\epsilon_{it}$ represents the error term. Standard errors are clustered on the level of the exiting GP. We do not include further covariates apart from the essential time and individual fixed effects.

To interpret $\beta_{j}$ as causal effects for the periods j>0, we need to assume that if the GP did not exit and continued practicing after t=0, the outcomes of their patients would not have changed (apart from a *common trend* arising, for instance, due to aging). This is the common trend assumption of the two‐way fixed effects literature (see, e.g., Callaway and Sant'Anna 2021). Although this assumption is inherently untestable, one implication is that if it holds, there should not be a trend in the outcomes in the quarters before GP's exit can be anticipated. Hence, $\beta_{j}$ must be zero for j≤a, where a is the reference period. In periods between a and 0, we allow for an anticipation of the GP's exit (for instance, by switching GPs before the exit). In our setting, for all outcomes and throughout all specifications, we do not find evidence for a deviation from a common trend five quarters before the GP exit. As we set our reference period six quarters before the exit (a=−6), we can identify causal effects with this treatment anticipation assumption (see Callaway and Sant'Anna 2021) because we compare post‐treatment outcomes with the reference period, which is not contaminated by anticipation effects.

Notice that in our setting, every patient experiences an exit of their GP at some point. Hence, we essentially compare changes in the respective outcome of an individual whose GP exited in a year to corresponding changes of another individual whose GP did not yet exit by that year. While dropping the non‐exit sample is beneficial as all patients share potentially unobserved characteristics that made them choose a soon‐exiting GP in the first place, causal inference can be problematic with two‐way fixed effects. This is because dynamic treatment effects could interfere with the implicit control group of already‐treated individuals (see Sun and Abraham 2021; De Chaisemartin and d'Haultfoeuille 2020; Goodman‐Bacon 2021). To avoid such contamination between dynamic treatment effects and an implicit control group, we apply the estimators of Borusyak, Jaravel, and Spiess (2023) and Sun and Abraham (2021). As an anticipation of the results, it shows that the results are pretty similar between these new and the conventional estimation methods, which is potentially due to a somewhat stable evolution of the treatment effects after five quarters past GP exit.

## 5 Results

### 5.1 Main Results

Before presenting the event‐study results for health care utilization in the form of primary care, specialist, and hospital services and diagnoses of chronic conditions, tests, and prescriptions, we first investigate the impact of the exit on access to primary care: Time until patients consult a new GP and the effects on the number of patients (with the same insurance) of the GP.

#### 5.1.1 Healthcare Access

**FIGURE 1:**
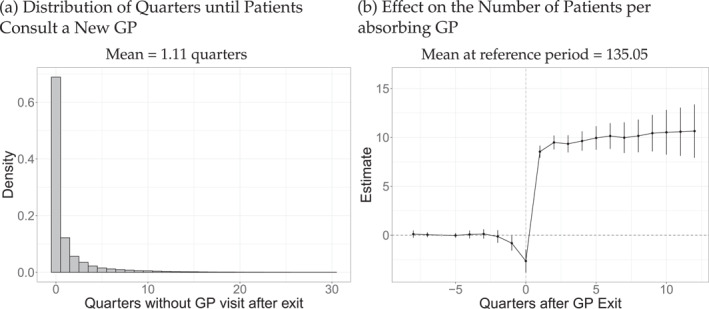
Practice characteristics. Number of observations in (a) 383,502 patients, in (b) 1,089,466 observations of 31,622 physicians.

**FIGURE A2:**
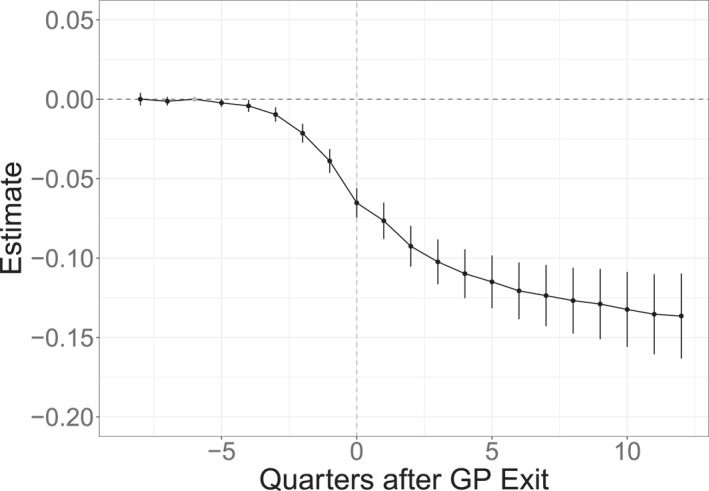
Effect on the share of patients in single practices. Number of observations: 15,340,080 observations of 383,502 patients. Mean at reference period = 0.68.

Patients find a new GP reasonably quickly, as seen in Figure 1a, which shows the distribution of the time it takes patients to find a new GP. Almost 70% of patients see a GP in the quarter after their previous GP leaves. Compared to their old practice, patients switch to a group practice more often, as shown in Figure A2. Considering the number of patients per GP (i.e., insurees in our claims data), Figure 1b reveals positive effects.^8^ The average number of patients of the new physicians in the sixth quarter before the exit is 135. After the exit, absorbing physicians treat about 10 more patients, corresponding to an increase of 7.4%. Hence, the exit of the old GP causes patients to attend more crowded practices, where it is likely that the GP has less time for each patient. Together with the almost‐stylized fact of a high general GP workload, the high work‐related stress levels of GPs (Siegrist et al. 2010; Voltmer et al. 2024), which is particularly increased among younger physicians and for consultations with new patients (von dem Knesebeck et al. 2019), this implies that GP exits pose meaningful frictions for the accessibility of primary care.

#### 5.1.2 Health Care Utilization

**FIGURE 2:**
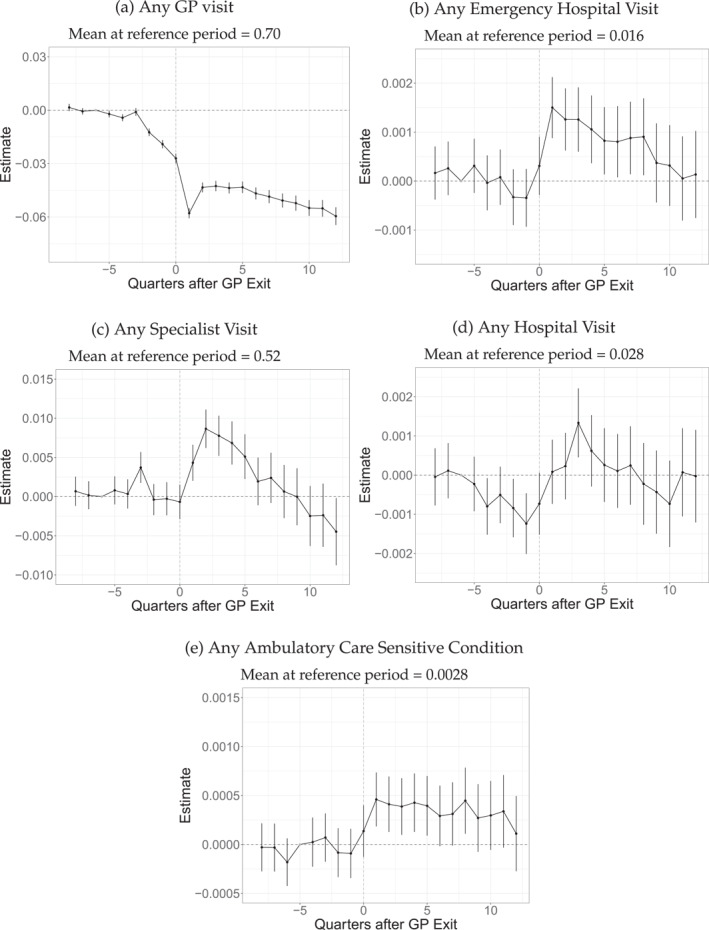
Event study results on healthcare utilization. The graph depicts the event‐study estimates (the $\beta_{j}^{ES}$ from Equation (1)). Vertical lines indicate 95% confidence intervals. The observation number is 15,340,080, with 383,502 unique patients and 7376 different leaving physicians (GP). GP visits include all visits to GPs. Emergency Hospital Visits include all hospital stays without a referral from a physician. Specialist visits include all visits to physicians without GPs, gynecologists, or pathologists. Hospital visits include all hospital stays, excluding births and emergency hospital visits. Ambulatory Care Sensitive Conditions include all hospitalizations related to ACSC, as defined by Albrecht and Sander (2015).

**FIGURE A3:**
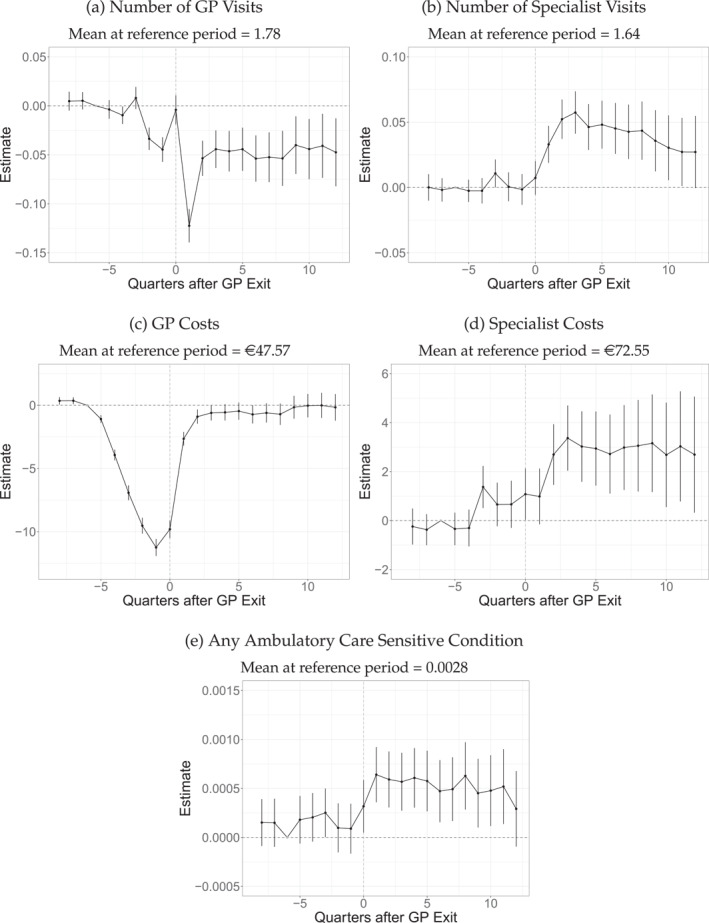
Additional results on health care utilization. The graph depicts the event‐study estimates (the $\beta_{j}^{ES}$ from Equation (1)). Vertical lines indicate 95% confidence intervals. The observation number is 15,340,080, with 383,502 unique patients and 7376 different leaving physicians (GP). GP visits include all visits to GPs. Specialist visits include all visits to physicians without GPs, gynecologists, or pathologists. The number of visits is the number of unique days services were billed to the health insurance. Costs are based on the *fee schedule* (EBM) points. Ambulatory Care Sensitive Conditions include all hospitalizations related to ACSC, as defined by Albrecht and Sander (2015).

Next, we turn to the effects on healthcare utilization, using our main specification in Equation (1). Panel 2a in Figure 2 presents the results for any GP visit in the corresponding quarter as an outcome. We plot the point estimates for $\beta_{j}^{ES}$ along the corresponding time since the patients' GP exited, which we confine from −8 to 12 (i.e., 2 years before until 3 years after the exit). The vertical lines around the estimate indicate the 95% confidence intervals. These estimates exhibit at least three interesting and essential features. First, no clear pretrends are visible up to three‐quarters before the exit, including our reference quarter −6. Second, we see an anticipation phase two quarters before the exit until the GP drops out. Here, the probability of visiting a GP decreases by one additional percentage point every quarter. Individuals know that their traditional GP will exit and stop visiting them. Finally, the dynamic effects of the exit become visible. In the quarter after the GP exit, the probability of consulting a GP directly reduces by about six percentage points (pp). In the following quarter, this negative effect slightly attenuates to four pp below the pre‐anticipation level, before the effects persist on this level in subsequent periods. We take this as evidence that the exit of the physician results in a permanent decrease in the probability of seeing a GP, which is—given the probability of seeing a GP of 70% in the reference period six quarters before the exit—of significant size. This relative decrease of 5%–6% is considerably larger than the −3% found for Denmark (Simonsen et al. 2021) and more in line with results for the US from Staiger (2022) and Zhang (2022) with −5.8% and −4.7% respectively. We present the effects for the intensive margin (number of visits) and total costs of GP visits in Figure A3a,c (showing that the number of visits decreases persistently, whereas costs remain largely unaffected).

Next, we investigate the spillovers to other forms of healthcare utilization. Figure 2b depicts the results for emergency hospital stays. These are defined as those hospital stays that were not referred by a physician, and thus represent a substitution of GP services that is, independent of GPs. Here, the results lack pretrends and anticipatory effects. However, in the year following the GP exit, the share of individuals with emergency hospital visits is about 0.1 pp higher than before, a relative increase of 6%, compared to the baseline level of 1.6%. We take this as evidence for a short to medium‐term substitution of GP services with hospital services.

Another substitution form might be specialist services, especially since GPs do not have a formal gatekeeping function in Germany. However, from anecdotal evidence and personal experience, we know that it is tough to make an appointment with a specialist without the referral of a GP (when making an appointment, one is often asked for a referral, see Werbeck, Wübker, and Ziebarth 2021). Therefore, specialist services present a form of substitution that is, dependent on GP services. The probability of seeing a specialist in Figure 2c shows no clear pretrends, including the quarters where we detect anticipatory effects for GP visits. We observe an increase in the probability of seeing a specialist by 0.75 pp in the second quarter after the exit. However, given the baseline value of 52%, the relative magnitude of this effect (1.4%) seems negligible. This small effect is followed by a steady decline, leading to null estimates in the medium term. Again, we present the effects on the intensive margin and on the costs of specialist visits in Figure A3 (Panels b and d, showing a significant and persistent increase of about 3% and 4%, respectively). In the literature, substitution effects depend on organizational structures. Simonsen et al. (2021) finds evidence of a reduction of specialist visits in Denmark (where GPs serve a gatekeeping function), whereas Zhang (2022) and Sabety, Jena, and Barnett (2021) find evidence for the increased use of specialist services for the US medicare population of older individuals. For Switzerland (Bischof and Kaiser 2021), a relative increase of 11% can be observed. Compared to this, our results show a small increase in the use of specialist services, which is still evidence for substituting GP services with specialist services.

The results for non‐emergency hospital visits are depicted in Figure 2d. These include all hospital visits that were referred by a physician and therefore also indicate substitution that is, initiated by the GP. All in all, these results appear relatively noisy. However, the probability of staying in a hospital decreases by about 0.1 pp in the year before the exit. Three quarters after the exit, it increases temporarily by 0.1 pp. Comparing this to an individual's average hospital share in the reference quarter of about 3%, these short‐term effects still represent a relative change of more than 3%. The decrease before the closure is most likely a result of the reduced GP visits, which prevent GPs from referring their patients in need to a hospital. Our results align with those of Zhang (2022), finding a 3% increase in the probability of hospitalizations, whereas Staiger (2022) does not find any effect for the general population. The reason for the increase in hospitalizations after the decrease is unclear: is it a catch‐up effect of missed hospitalization before the closure, or is it related to the new physicians' practice styles?

To shed light on this aspect, we next assess hospitalizations with ambulatory care‐sensitive conditions, which could have been prevented with adequate ambulatory care. For instance, these conditions include hospitalizations for asthma and diabetes or hospitalizations for chronic ischemic heart diseases that do not include surgical operations (Albrecht and Sander 2015). The results are depicted in Figure 2e.^9^ No pretrends or anticipatory effects are visible. While the estimates exhibit a clear jump in the quarter after the exit, the share of individuals hospitalized with an ambulatory care‐sensitive condition increases by almost 0.05 pp. Given the baseline value of 0.3%, these estimates represent a relative increase of 16%. However, this effect is only evident in the short run and moves toward zero 1.5 years after the exit. This indicator of care quality is only used by Zhang (2022), finding no effect.

In sum, our results indicate that the exit of the GP disrupts the utilization of primary care. In response, individuals partly switch to hospital services. We find evidence that new GPs increasingly refer patients to specialists and hospitals, but only in the short and medium term. Moreover, we find evidence that ambulatory care quality decreases, as evidenced by increased avoidable hospitalizations. However, as Figure A7 shows, these effects do not translate to an increase in mortality.

#### 5.1.3 Diagnoses

**FIGURE 3:**
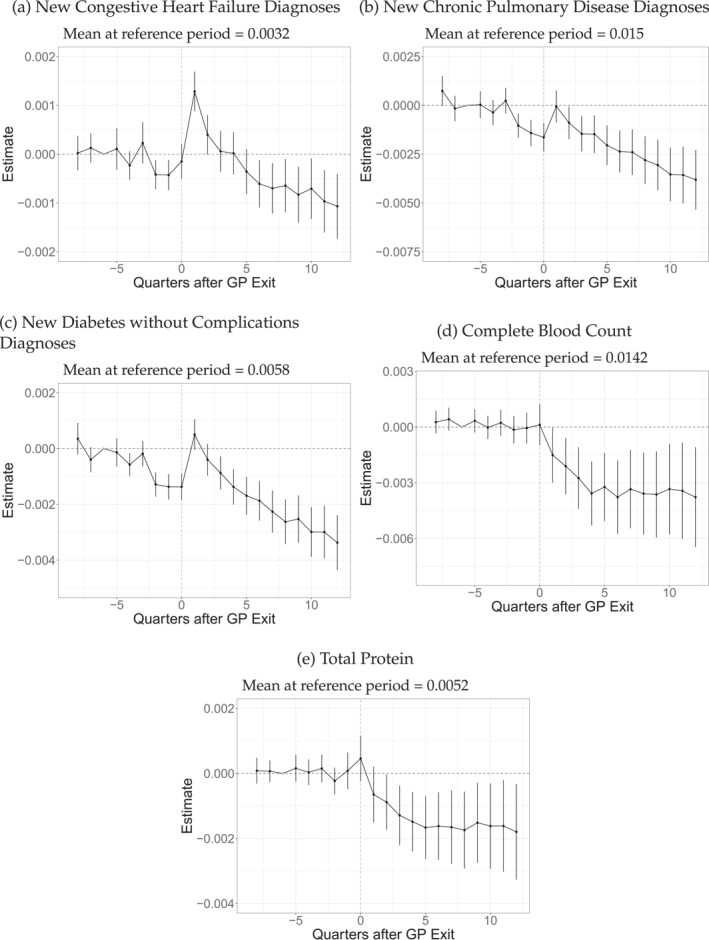
Event study results on specific diagnoses. The graph depicts the event‐study estimates (the $\beta_{j}^{ES}$ from Equation (1)). Vertical lines indicate 95% confidence intervals. The observation number is 15,340,080, with 383,502 unique patients and 7376 different leaving physicians. The outcome variable is equal to one in the first quarter in the observational period that the respective disease (as defined by the Chalson Comorbidity Index; Charlson et al. 1987) is diagnosed and 0 else. Complete Blood Count as defined by EBM No. 32122. Total Protein as defined by EBM No. 32056.

**FIGURE A4:**
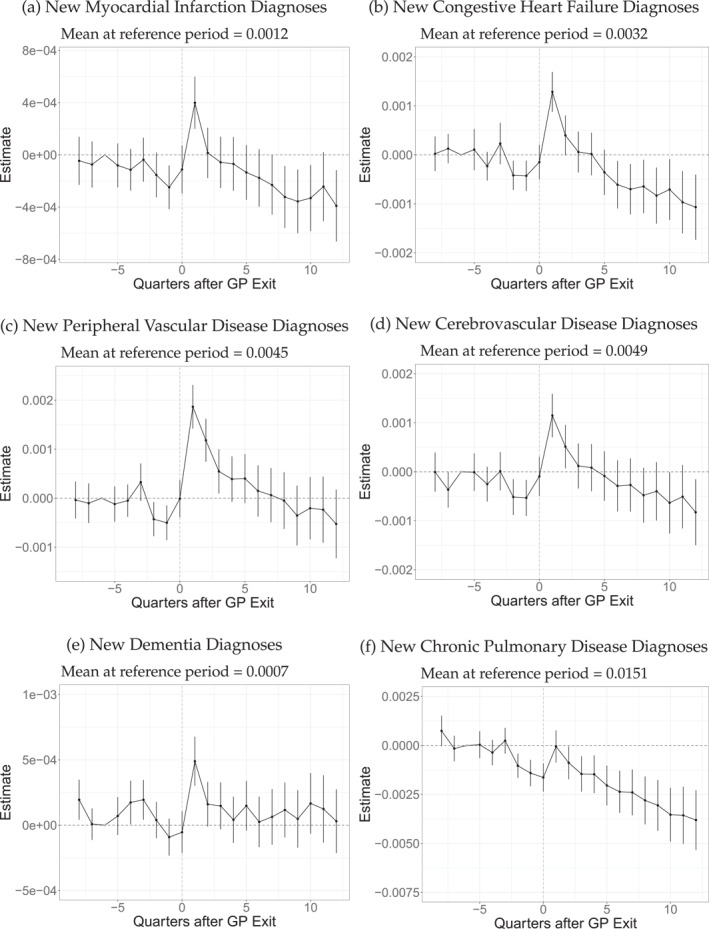
Additional results on specific diagnoses. The graph depicts the event‐study estimates (the $\beta_{j}^{ES}$ from Equation (1)). Vertical lines indicate 95% confidence intervals. The observation number is 15,340,080, with 383,502 unique patients and 7376 different leaving physicians. The outcome variable is equal to one in the first quarter in the observational period that the respective disease (as defined by the Chalson Comorbidity Index; Charlson et al. 1987) is diagnosed and 0 else.

**FIGURE A5:**
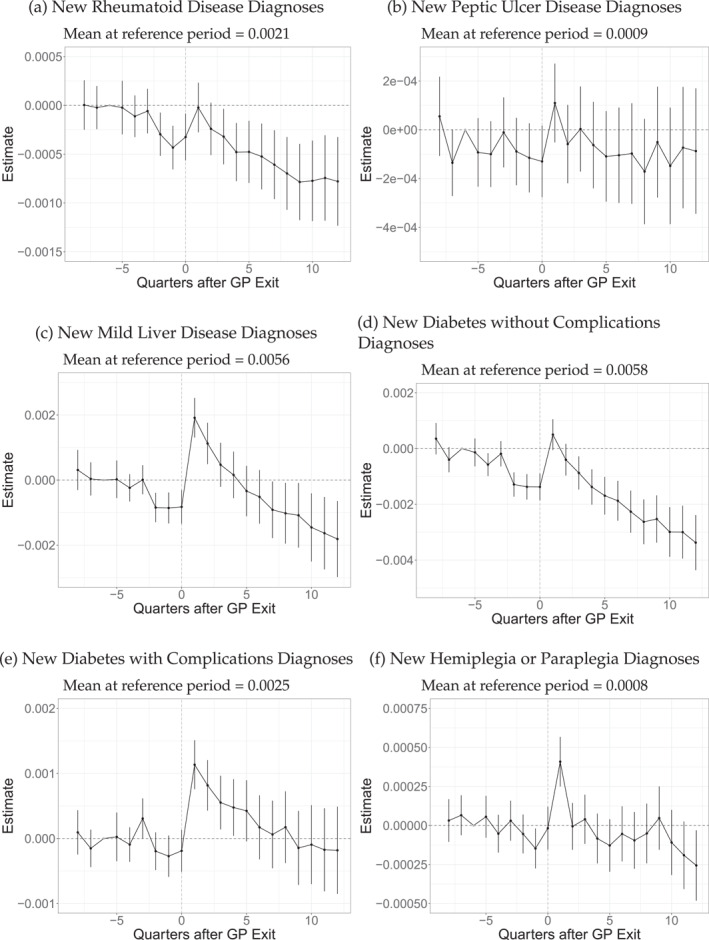
Additional results on specific diagnoses—cont. The graph depicts the event‐study estimates (the $\beta_{j}^{ES}$ from Equation (1)). Vertical lines indicate 95% confidence intervals. The observation number is 15,340,080, with 383,502 unique patients and 7376 different leaving physicians. The outcome variable is equal to one in the first quarter in the observational period that the respective disease (as defined by the Chalson Comorbidity Index; Charlson et al. 1987) is diagnosed and 0 else.

**FIGURE A6:**
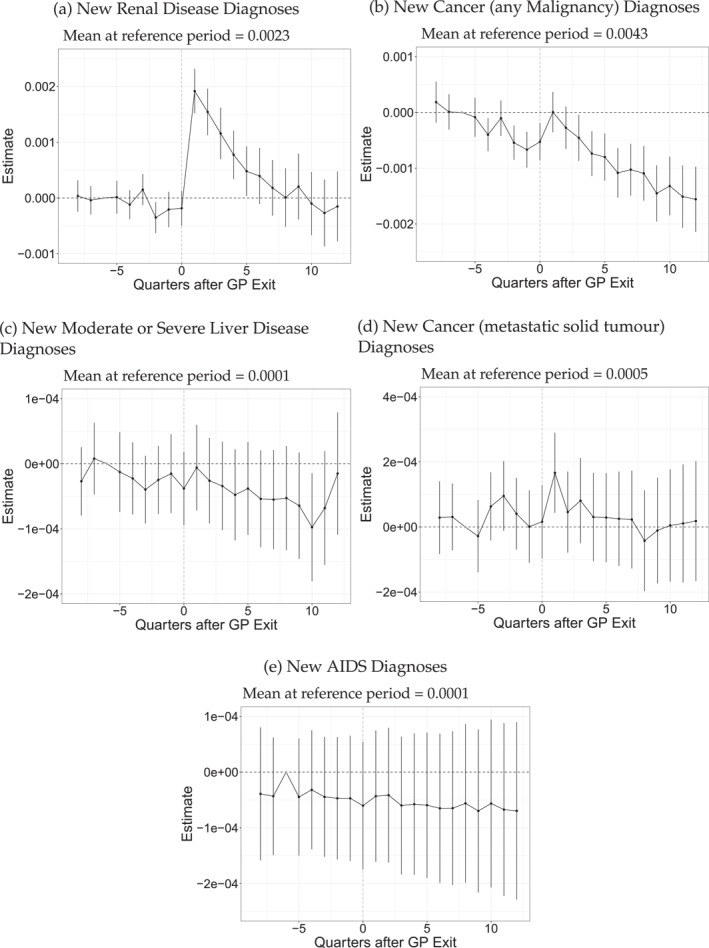
Additional results on specific diagnoses—cont. The graph depicts the event‐study estimates (the $\beta_{j}^{ES}$ from Equation (1)). Vertical lines indicate 95% confidence intervals. The observation number is 15,340,080, with 383,502 unique patients and 7376 different leaving physicians. The outcome variable is equal to one in the first quarter in the observational period that the respective disease (as defined by the Chalson Comorbidity Index; Charlson et al. 1987) is diagnosed and 0 else.

**FIGURE A7:**
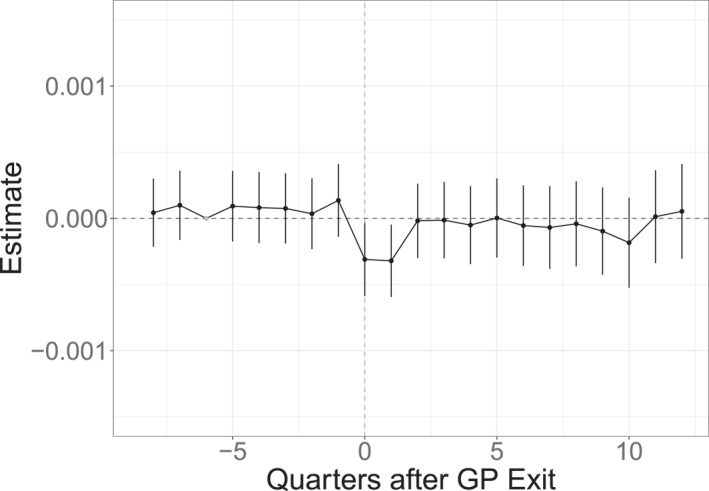
Event study results on mortality. The graph depicts the event‐study estimates (the $\beta_{j}^{ES}$ from Equation (1)). Vertical lines indicate 95% confidence intervals. Mean at reference period = 0.004. The observation number is 18,593,392, with 418,582 unique patients and 7376 different leaving physicians.

We now investigate the impact of GP exits on diagnoses as defined by the Charlson Comorbidity Index (Charlson et al. 1987). We present results for the diagnoses of congestive heart failure, chronic pulmonary disease, and diabetes in Figure 3 and show results for all remaining diagnoses in Appendix A for completeness (Figures A4, A5, A6). Contrary to the values depicted in Table 1, we use a dummy, indicating the first quarter in which they are documented by any physician in the observational period. The results for all these diagnoses exhibit a similar pattern: pretrends are absent before quarter three, with a slight dip before the exit. New diagnoses spike in the first quarter after the exits (even if this spike is not always greater than zero), after which they commonly decline (12 of 20 assessed diagnoses are negatively affected, with the remaining diagnoses being unaffected). Congestive heart failure detection, for example, increases by 0.14 pp (Figure 3a), compared to a baseline of 0.3%, which translates to a relative increase of 46%. The most prevalent disease is Chronic Pulmonary Disease, with 10% of individuals diagnosed in the reference period (See Table 1). Although there is a decrease in detection before the exit, the detection rate rises to the pre‐treatment level in the quarter after the exit, after which it declines continuously (Figure 3b). We observe a similar trend for diabetes diagnoses (Figure 3c). The decline of most diagnoses over time is most likely a result of the decreased GP visits. It indicates a negative impact of the exit since the negative estimates most likely indicate missed diagnoses.

We supplement the findings for diagnoses with evidence for diagnostic testing in Figure 3d,e, presenting results for blood tests in the form of a complete blood count and total protein tests, respectively. As can be seen, adverse effects of about −0.4 pp exist for complete blood counts and −0.15 pp for total protein tests, which are of significant size, compared to the respective baseline values of 1.4% and 0.5%. The decline is persistent and follows the pattern of GP visits in general (Figure 2a). This indicates that the spikes observed for diagnoses result from new GPs recording existing diagnoses. In principle, simultaneously decreased diagnostic testing and filed diagnoses of the chronic conditions could suggest that new GPs have received past medical records of patients (which is possible, see Section 2), such that new tests and diagnoses are superfluous. However, the substantial long‐term decrease in diagnoses and the reduction in GP contacts make it appear more likely a general decline in healthcare quality is the driver behind the results for tests and diagnoses. Hence, this is an important finding, which is undocumented in the literature so far.^10^

#### 5.1.4 Prescriptions

**FIGURE 4:**
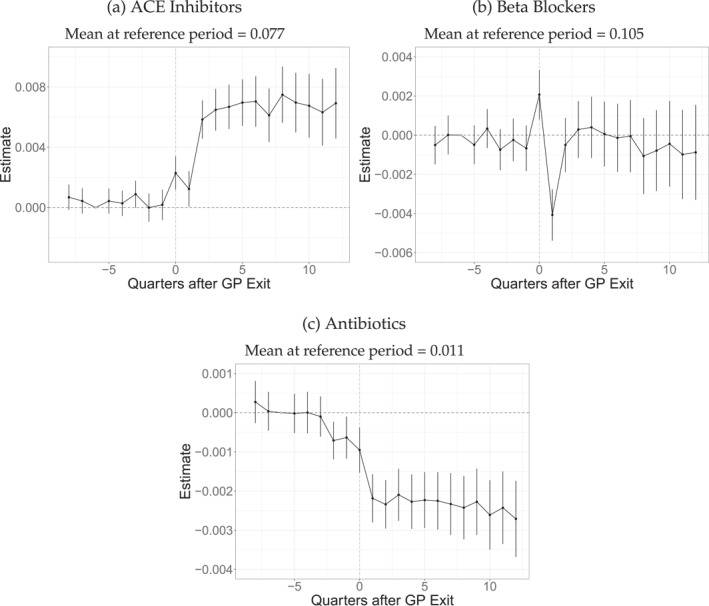
Event study results on prescriptions. The graph depicts the event‐study estimates (the $\beta_{j}^{ES}$ from Equation (1)). Vertical lines indicate 95% confidence intervals. The observation number is 15,340,080, with 383,502 unique patients and 7376 different leaving physicians. ACE Inhibitors include all prescriptions with ATC C09a and C09b. Beta Blockers include all prescriptions with ATC C07. Antibiotics include all prescriptions with ATC J01.

**FIGURE A8:**
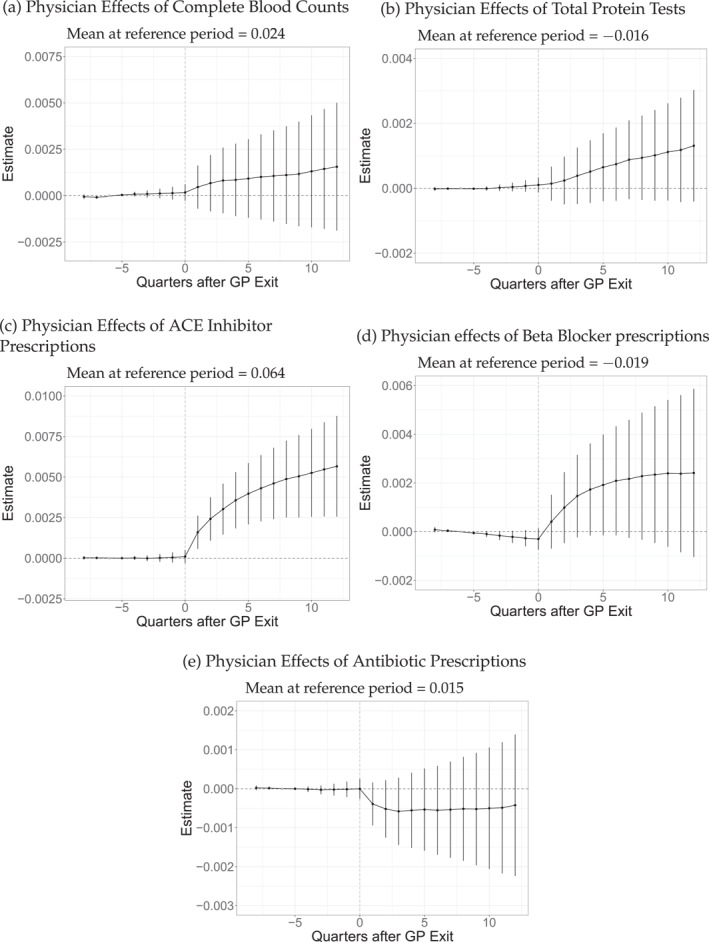
Event study results on physician characteristics. The graph depicts the event‐study estimates (the $\beta_{j}^{ES}$ from Equation (1)). Vertical lines indicate 95% confidence intervals. The observation number is 15,340,080, with 383,502 unique patients and 7376 different leaving physicians.

We also investigate whether the exit has effects on specific prescriptions. Figure 4a presents results for the prescription of ACE Inhibitors, while Figure 4b presents results for Beta Blockers. With ACE Inhibitor prescriptions, we observe increased prescriptions after the exit of 0.7 pp. Taking the baseline of 7.7% into account, this effect is meaningful. On the other hand, we observe no effects on the prescription of Beta Blockers in the long run. We investigate the effects on prescriptions of Antibiotics in Figure 4c. We observe a persistent decrease of about 0.25 pp compared to a baseline of 1%. Figure A8 shows results for physician effects for test and prescriptions. We find evidence for physician effects only for ACE Inhibitors. In total, slight evidence favors the interpretation of absorbing GPs being more prone to prescribe according to recent medical knowledge than leaving GPs. On the other hand, prescribing ACE inhibitors may be a purely preventive measure that does not require specific knowledge about the patient's true health.

### 5.2 Effect Heterogeneities and Robustness Checks

We now explore the heterogeneity of our results. To keep these results traceable, we abstain from showing event‐study plots and estimate more aggregate results, distinguishing our estimates between four phases. These phases capture the important properties of all the presented results: The pretrends (in quarters −8 and −7), the anticipation phase (at most from quarters −5 to −1), the first year (quarters 0–3), the medium run (quarters 4–12) and the long run (quarters 13 and upwards) for which it should be kept in mind that the sample is not balanced. In particular, we split the sample by gender, age (with a median split), between rural and urban counties, and patient comorbidities (determined by any diagnosis of the Charlson Comorbidity Index in any of the eight quarters before the GP exit), as well as whether patients were treated by a physician in single practice or group practice. Table 2 reports estimates of this regression on healthcare utilization without pretrends (as they are negligible), the anticipation phase, and long‐term effects (note that coefficients are scaled by 100). The complete results for all outcomes are presented in Supporting Information S1. Shifting the focus toward the new practices, Tables S18–S20 show heterogeneities as defined by the characteristics of the new practices. The first split is between patients who visit a single practice after the exit and those who visit a group practice. The second split is between patients who see a GP with more patients than their leaving GP versus those that see a GP with fewer patients. Together with the point estimates, the tables provide the standard error in parentheses and the adjusted *p*‐value in brackets. The latter is adjusted for multiple hypothesis testing, following Benjamini and Hochberg (1995), taking into account that for each subsample, 34 estimates are shown overall.

**TABLE 2:** Effects by specific sub‐groups (coefficients multiplied by 100).

	Any GP visit		Any specialist visit		Any hospital visit		Observations
	First year	Med. run	First year	Med. run	First year	Med. run	
Complete sample	−3.2338	−2.9579	0.8842	0.8809	0.0589	0.0729	15,340,080
	(0.1142)	(0.1321)	(0.0972)	(0.1171)	(0.0340)	(0.0383)	
	[0.0000]	[0.0000]	[0.0000]	[0.0000]	[0.1009]	[0.0750]	
Gender							
Female	−2.9655	−2.7497	0.8841	0.7833	0.0787	0.0553	9,231,320
	(0.1271)	(0.1496)	(0.1207)	(0.1443)	(0.0438)	(0.0496)	
	[0.0000]	[0.0000]	[0.0000]	[0.0000]	[0.0946]	[0.2906]	
Male	−3.6526	−3.2936	0.8708	1.0046	0.0275	0.0969	6,108,760
	(0.1558)	(0.1819)	(0.1410)	(0.1696)	(0.0514)	(0.0583)	
	[0.0000]	[0.0000]	[0.0000]	[0.0000]	[0.6300]	[0.1428]	
Individuals							
Older	−2.0263	−1.9438	1.3810	1.4174	0.1377	0.1205	7,794,800
	(0.1320)	(0.1548)	(0.1295)	(0.1545)	(0.0547)	(0.0625)	
	[0.0000]	[0.0000]	[0.0000]	[0.0000]	[0.0184]	[0.0799]	
Younger	−4.5003	−4.0670	0.3716	0.3204	−0.0174	0.0313	7,545,280
	(0.1522)	(0.1795)	(0.1326)	(0.1595)	(0.0375)	(0.0416)	
	[0.0000]	[0.0000]	[0.0143]	[0.0797]	[0.7285]	[0.5484]	
Area							
Rural	−3.2336	−2.9912	1.1587	1.0845	0.0223	0.0382	4,797,560
	(0.1898)	(0.2110)	(0.1742)	(0.2033)	(0.0611)	(0.0695)	
	[0.0000]	[0.0000]	[0.0000]	[0.0000]	[0.7622]	[0.6610]	
Urban	−3.2561	−3.0176	0.7595	0.7410	0.0736	0.0868	9,839,000
	(0.1413)	(0.1668)	(0.1184)	(0.1447)	(0.0416)	(0.0470)	
	[0.0000]	[0.0000]	[0.0000]	[0.0000]	[0.1018]	[0.0945]	
Comorbidities							
With	−2.8005	−2.9488	1.0526	0.6386	0.0158	−0.0561	8,952,560
	(0.1313)	(0.1508)	(0.1214)	(0.1464)	(0.0509)	(0.0575)	
	[0.0000]	[0.0000]	[0.0000]	[0.0000]	[0.8034]	[0.3729]	
Without	−3.5061	−2.4500	0.8641	1.5377	0.1365	0.2721	6,387,520
	(0.1582)	(0.1876)	(0.1421)	(0.1685)	(0.0383)	(0.0434)	
	[0.0000]	[0.0000]	[0.0000]	[0.0000]	[0.0005]	[0.0000]	
Practice							
Single	−3.3523	−2.8115	0.9657	1.1544	0.1309	0.1550	9,699,160
	(0.1436)	(0.1661)	(0.1212)	(0.1449)	(0.0429)	(0.0487)	
	[0.0000]	[0.0000]	[0.0000]	[0.0000]	[0.0039]	[0.0029]	
Group	−3.0188	−3.1522	0.7511	0.4383	−0.0605	−0.0650	5,640,920
	(0.1752)	(0.2128)	(0.1571)	(0.1921)	(0.0551)	(0.0618)	
	[0.0000]	[0.0000]	[0.0000]	[0.0383]	[0.3508]	[0.3555]	

*Note:* Standard errors in parentheses. Adjusted *p*‐values following Benjamini and Hochberg (1995) with *m* = 34 (the number of presented point estimates per subsample), applied for each sub‐sample separately, in brackets. *First Year* = event times 0–3, *Medium Term* = event times 4–12; *Complete Sample* = all observations, *Female* = only females, *Male* = only males, *Older Individuals* = individuals born before or in the median birth year of 1957, *Younger Individuals* = individuals born after the median birth year of 1957, *Rural* = individuals living in a county, where the share of inhabitants that live in municipalities with more than 150 Inhabitants per km^2^ is less than 75%, *Urban* = Individuals living in a county, where the share of inhabitants that live in municipalities with more than 150 Inhabitants per km^2^ is more than 75%, *Comorbidities* = individuals, that received at least one diagnosis of the Charlson Comorbidity Index by a Physician in the eight quarters before the exit of their GP, *Single Practice* = leaving physician practiced in a single practice, *Group Practice* = leaving physician practiced in a group practice.

Generally, there do not seem to be any substantial gender differences or differences between individuals living in urban or rural areas. The latter is especially surprising, as one would expect that it is more difficult for patients to find a new GP in rural areas. One possible explanation is that differences between urban and rural areas are irrelevant in the *extensive* margin. Even though patients face longer driving times in rural areas, they might still manage to attend appointments. However, they might prefer (or are able) to make fewer visits to their new GPs because of the distance. This explains why we see differences in the *intensive* margin, the number of GP visits per quarter (see Table S13). In contrast, age appears to be an essential factor, with older individuals having higher levels of healthcare utilization after the exit. This could be due to a higher healthcare elasticity of younger relative to older individuals, which could arise as the implicit price for healthcare utilization increases because of the exit (in the form of decreased accessibility). A split by comorbidities also supports this, as comorbid individuals have a lower healthcare elasticity. Considering the differences between leaving GPs who work in a group practice versus those working in a single practice, there do not appear to be differences in GP utilization. In contrast, patients of single‐practice physicians are referred to specialists and hospitals more often after the exit. Table S18 supports the idea that fuller practices after the exit contribute to the reduction in GP visits. Patients whose new GP treats more patients than the leaving GP are three percentage points less likely to visit a GP, compared to 1.5 percentage points for patients who see GPs with fewer patients. Patients in group practices are more likely to receive ACE inhibitor prescriptions. This might be explained by the fact, that younger GPs prefer to be employed in group practices and are more likely to prescribe according to recent medical knowledge.

#### 5.2.1 Robustness

**FIGURE A9:**
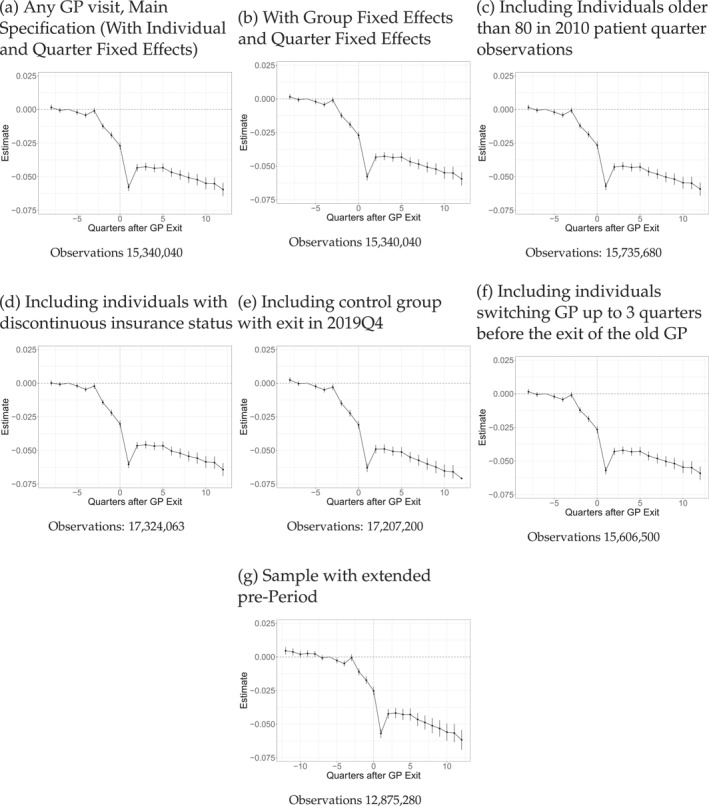
Event study results on any GP visit, robustness tests. The graph depicts the event‐study estimates (the $\beta_{j}^{ES}$ from Equation (1)). Vertical lines indicate 95% confidence intervals. Observations are on the patient‐quarter level.

We test for several types of robustness. In Figure A9, we provide results for different model specifications using our main outcome variable of any GP visit in a given quarter. Figure A9a is the main specification as used before. In Figure A9b, we use fixed effects for the leaving physician (instead of individual fixed effects). In Figure A9c, we also include all individuals older than 80 in 2010 (and surviving until 2019) in the estimation. In the main specification, we use only those individuals who are continuously insured from 2010 to 2019. We drop this restriction in Figure A9d, only conditioning on observing individuals from Event Times −8 to 12. We thereby include individuals who die or leave the insurance during the observational period. In Figure A9e, we include individuals whose GP exits in the last quarter of 2019 as a control group. Descriptive statistics for this sample can be found in Table S21. We set event time −6 as the reference period for the whole group. We do not use this specification as our main specification because using the *not yet treated* as a control group might bias the estimates as this may be selective regarding unobserved trends (violating the common trend assumption). We include individuals switching their GP up to three quarters before their GP resigns (Figure A9f). Lastly, Figure A9g presents results for patients whose GPs exit from 2013 to 2016, allowing to extend the pre‐event period to 12 quarters before the exit. All in all, none of the results differed meaningfully from our main specification. Thus, we conclude that the restrictions we used to define our main estimation sample did not significantly impact our results.

**FIGURE A10:**
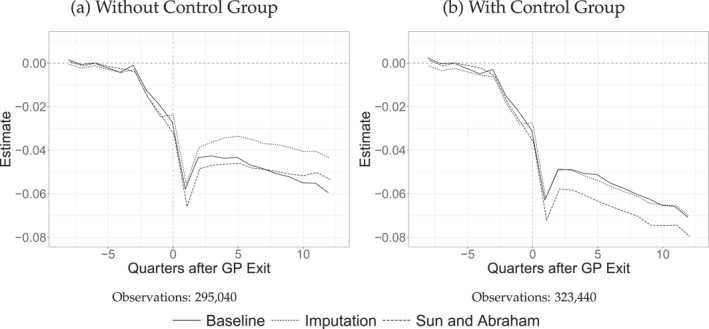
Event study results on any GP visit, robustness tests. *Baseline*: Estimation as described above. *Imputation*: Estimation using the Imputation estimator following Borusyak, Jaravel, and Spiess (2023). *Sun and Abraham*: Estimation following Sun and Abraham (2021). For computational purposes, we aggregated the observations first to the GP‐quarter level.

Figure A10 checks whether our results suffer from the potentially adverse consequences in two‐way fixed effects models (which may use already treated units as an implicit control group). For event study estimators, Sun and Abraham (2021), among others, draw attention to this important source of bias. We present estimates for our baseline model as described above (for computational purposes, we first aggregated the data on the quarter GP level) for the imputation estimator following Borusyak, Jaravel, and Spiess (2023), and for the approach described by Sun and Abraham (2021).^11^ In Figure A10a, we use our main specification without including a control group, while Figure A10b includes the same control group as Figure A9e. Although there are some minor differences between the estimates of the treatment effects, we argue that the more sophisticated estimators still virtually lead to the same interpretations as our baseline model.

## 6 Conclusion

We study the impact of a disruption in the patient‐physician relationship induced by GPs leaving the profession (for whatever reason). As we have argued, this causes a potential trade‐off. On the one hand, depending on the healthcare system, primary care accessibility is reduced, as all patients need to search for a new GP. On the other hand, finding a new GP may have beneficial consequences for healthcare quality: they are likely younger and, therefore, more informed about more up‐to‐date medical guidelines.

Our results show that the closure of a GP practice has a significant and long‐lasting negative impact on the probability of seeing a GP. At the same time, the effects on the number of visits and costs for GP services are less pronounced. There is evidence on substituting GP services with specialist and hospital services. However, this is only the case in the short run, and whether the extent of this substitution is large enough to make up for the reduction in primary care is questionable. This is further supported by the results for hospitalizations with ambulatory care‐sensitive conditions, which reveal a substantial and persistent negative impact of practice closures on patients. We observe an essential decrease in diagnoses of chronic conditions, suggesting that disruptions may have adverse consequences for the efficiency of the healthcare system. These negative consequences may ultimately be caused by fewer primary care visits, which in turn cause reduced diagnostic testing. Primary care may be reduced as the stock of patients of the new GPs is larger, preventing GPs from building good professional relationships with their patients.

Our results align more with those for the US (Sabety, Jena, and Barnett 2021; Staiger 2022; Zhang 2022) than for Denmark, where forced changes of physicians are much more organized (Simonsen et al. 2021). Although the healthcare systems of Switzerland and Germany are comparable, our results differ from Bischof and Kaiser (2021), who find an even more pronounced drop in GP visits. This, however, may result from the slight remaining differences in the healthcare system, particularly concerning insurance plans that limit provider choice and deductibles that may disincentivize seeking medical advice unless absolutely necessary. The healthcare systems of Austria and Germany seem to be more comparable. In line with Zocher (2024), we find a decrease in the probability of seeing a GP after the visit. However, we do not observe an increase in physician fees. Our results stand out from the literature since we are the first to document the negative consequences of GP exits for the healthcare market, as measured by missed diagnoses of chronic diseases.

Overall, our results can be explained by the fact that although the German healthcare system might be highly accessible in principle, the high general demand for services Blümel et al. (2020); OECD (2023) causes many GPs to be capacity‐constrained. Particularly, new patients suffer from GPs' resulting time constraints (von dem Knesebeck et al. 2019), which are pronounced in Germany (Siegrist et al. 2010; Voltmer et al. 2024) implying that GP resignations pose meaningful frictions to healthcare accessibility. Hence, our results reveal important insights into the importance of a good informal and long‐lasting relationship with the GP, particularly when there are capacity constraints. The upcoming demographic transition of GPs in Germany will cause many GPs to resign in the upcoming years while the demand for services will increase, it is essential to help patients and the GPs build such an informal relationship. Incentivizing GPs to offer additional consultation hours may be one short‐term solution. In the long run, expanding the GP workforce to help GPs increase the time per patient may be another.

Our study has several limitations. For instance, our estimation sample might not represent the general population, including the privately insured. Additionally, we conclude that the new GPs have less time for patients because absorbing GPs treats more patients. It would be optimal to present estimates for the effect on consultation or waiting times, but unfortunately, these are not included in our data. Still, we argue that the increase of the practice size by more than 7% is bound to affect the time per patient and quality in the relationship between GPs and patients—with which our results would be compatible. In line with this, we have limited information on the GPs—for example, we lack information on the GPs' experience, age, and the distance that patients travel to the practice. This information would help us further understand the drivers of the effects we observe.

Overall, our results paint a picture of the GP as Germany's primary coordinator of patient healthcare. Even though GPs in Germany do not serve as gatekeepers formally, they still fill this role informally. The GP's exit disrupts all forms of healthcare usage, and the limited access to primary care after the exit results in worse healthcare for patients overall.

## Conflicts of Interest

Dr. Westphal reports Grants from German Research Foundation (DFG), outside the submitted work.

## Supporting information

Supporting Information S1

## Data Availability

The authors have nothing to report.

## Endnotes

^1^
Note that we refer to GPs also as physicians unless otherwise declared.

^2^
The latter is open for permanent public employees and civil servants, self‐employed, and individuals earning more than the opt‐out threshold (€62,550 in 2020).

^3^
According to an answer from an inquiry we have sent to the Federal Association of Statutory Health Insurance Physicians.

^4^
There is the possibility that these GPs continue to serve privately insured patients, however, for SHI patients this still means a loss of the GP.

^5^
Gebührenordnungspositionen in German.

^6^
Note that the reasons for not being continuously insured are either dying or switching insurance in the observational period. While this might decrease the generalizability of our estimation sample, it ensures that neither drives our estimates. Generally, switching of insurance is rare, and there is no reason for it to be related to the exit of the GP.

^7^
We use the SOEP data of 2010, restricting it to individuals between 18 and 80 years old and using the population weights provided by the SOEP.

^8^
The underlying estimation for this figure follows the one in Equation (1) but is based on a panel of GPs that take over patients from exiting GPs.

^9^
Please note that we conservatively changed the reference period from −6 to −5 only in Figure 2e, as the outlier that would inflate the result. Figure A3e part from the higher level of the effect nothing else changes.

^10^
Zhang (2022) reports an increase in diagnoses of chronic conditions (+12%).

^11^
Since estimating these models is computationally more challenging, we aggregate the data on the level of the leaving physician and use the number of physician patients in a given quarter as the respective weight. This estimation lets us replicate our main results perfectly.

## Appendix A

### Practice Style of New Physicians

We try to shed light on changes in the general practice style of the GPs, which is independent of the specific patient. Hence, we have to disentangle the patient‐specific from the GP‐specific outcome. For this purpose, we follow the spirit of Simonsen et al. (2021) and first estimate outcome‐specific auxiliary regressions, where we regress each prescription and test in Figure 4 on patient, quarter, and physician dummies using data from 2010 to 2011, that is, the pre‐treatment periods.^12^ With patient‐fixed effects, the physician dummies are identified by patients who switch doctors and thereby achieve the goal of separating the two factors. We then take the estimates of the physician‐fixed effects as a proxy for the treatment style of the corresponding physician and assign them to each patient‐quarter observation in our sample. Finally, we use these estimated physician‐fixed effects as an outcome in the event study and present the results in Figure A8.
