# Supplementary material for: The Effects of Resigning GPs on Patient Healthcare Utilization and Some Implications for Health
Source: Health Econ. 2025 Jan 30;34(5):932–55. doi: 10.1002/hec.4941 (PMC11961348; doi:10.1002/hec.4941)
Supplement: Supplementary file 1 — Supporting Information S1 [file HEC-34-932-s001.pdf]

— For Online Publication: Supplementary Materials —

November 2024

Table S1: Results: Any GP Visits (coefficients multiplied by 100)

	Pretrend	Anticipation	First Year	Medium Term	Long Term	Observations
Complete Sample	−0.1351	−0.3803***	−3.2338***	−2.9579***	−4.0625***	15,340,080
	(0.0842)	(0.0843)	(0.1142)	(0.1321)	(0.1875)	
Female	−0.0644	−0.3409***	−2.9655***	−2.7497***	−3.8851***	9,231,320
	(0.0976)	(0.0942)	(0.1271)	(0.1496)	(0.2102)	
Male	−0.2348*	−0.4444***	−3.6526***	−3.2936***	−4.3611***	6,108,760
	(0.1248)	(0.1230)	(0.1558)	(0.1819)	(0.2471)	
Older Individuals	−0.0305	0.0050	−2.0263***	−1.9438***	−3.2316***	7,794,800
	(0.0982)	(0.0976)	(0.1320)	(0.1548)	(0.2257)	
Younger Individuals	−0.2277*	−0.7806***	−4.5003***	−4.0670***	−5.0256***	7,545,280
	(0.1225)	(0.1191)	(0.1522)	(0.1795)	(0.2378)	
Rural	0.0595	−0.3545**	−3.2336***	−2.9912***	−4.1037***	4,797,560
	(0.1451)	(0.1503)	(0.1898)	(0.2110)	(0.3141)	
Urban	−0.2001*	−0.4198***	−3.2561***	−3.0176***	−4.1499***	9,839,000
	(0.1050)	(0.1030)	(0.1413)	(0.1668)	(0.2318)	
Comorbidities	−0.1298	0.0426	−3.0358***	−3.6705***	−5.5322***	7,874,760
	(0.0975)	(0.0987)	(0.1359)	(0.1552)	(0.2274)	
No Comorbidities	−0.1359	−0.8119***	−3.4058***	−2.1478***	−2.4482***	7,465,320
	(0.1234)	(0.1172)	(0.1496)	(0.1773)	(0.2332)	
Single Practice	−0.0617	−0.5845***	−3.3523***	−2.8115***	−3.4574***	9,699,160
	(0.1050)	(0.1050)	(0.1436)	(0.1661)	(0.2247)	
Group Practice	−0.2598*	−0.0246	−3.0188***	−3.1522***	−4.9847***	5,640,920
	(0.1350)	(0.1399)	(0.1752)	(0.2128)	(0.3179)	

Notes: Standard Errors in Parentheses. *Pretrend* = Event Times -8 to -7, *Anticipation* = Event Times -5 to -1, *First Year* = Event Times 0 to 3, *Medium Term* = Event Times 4 to 12, *Medium Term* = Event Times 13 and greater; *Complete Sample* = All Observations, *Female* = Only females, *Male* = Only males, *Older Individuals* = Individuals born before or in the median birth year of 1957, *Younger Individuals* = Individuals born after the median birth year of 1957, *Rural* = Individuals living in a county, where the share of inhabitants that live in municipalities with more than 150 Inhabitants per km^2 is less than 75%, *Urban* = Individuals living in a county, where the share of inhabitants that live in municipalities with more than 150 Inhabitants per km^2 is more than 75%, *Comorbidities* = Individuals, that received at least one diagnosis of the Charlson Comorbidity Index by a Physician in the eight quarters before the exit of their GP, *Single Practice* = Leaving physician practiced in a single practice, *Group Practice* = Leaving physician practiced in a group practice. .

Table S2: Results: Number of GP Visits

	Pretrend	Anticipation	First Year	Medium Term	Long Term	Observations
Complete Sample	0.0042	−0.0149***	−0.0515***	−0.0388***	−0.0520***	15,340,080
	(0.0040)	(0.0043)	(0.0068)	(0.0091)	(0.0126)	
Female	0.0025	−0.0166***	−0.0443***	−0.0373***	−0.0503***	9,231,320
	(0.0046)	(0.0048)	(0.0074)	(0.0101)	(0.0137)	
Male	0.0068	−0.0125**	−0.0627***	−0.0418***	−0.0555***	6,108,760
	(0.0053)	(0.0058)	(0.0083)	(0.0108)	(0.0151)	
Older Individuals	0.0113**	−0.0089	−0.0175*	−0.0170	−0.0362**	7,794,800
	(0.0057)	(0.0060)	(0.0092)	(0.0124)	(0.0168)	
Younger Individuals	−0.0036	−0.0197***	−0.0837***	−0.0597***	−0.0728***	7,545,280
	(0.0045)	(0.0045)	(0.0070)	(0.0091)	(0.0130)	
Rural	−0.0019	−0.0250***	−0.0686***	−0.0632***	−0.0916***	4,797,560
	(0.0068)	(0.0074)	(0.0112)	(0.0145)	(0.0196)	
Urban	0.0065	−0.0121**	−0.0451***	−0.0300***	−0.0373**	9,839,000
	(0.0050)	(0.0054)	(0.0084)	(0.0115)	(0.0158)	
Comorbidities	0.0051	$2e-04$	−0.0650***	−0.0861***	−0.1263***	7,874,760
	(0.0058)	(0.0063)	(0.0099)	(0.0129)	(0.0177)	
No Comorbidities	0.0032	−0.0303***	−0.0357***	0.0142*	0.0320***	7,465,320
	(0.0041)	(0.0040)	(0.0059)	(0.0080)	(0.0109)	
Single Practice	0.0072	−0.0286***	−0.0475***	−0.0267**	−0.0294**	9,699,160
	(0.0049)	(0.0051)	(0.0082)	(0.0110)	(0.0148)	
Group Practice	−0.0018	0.0088	−0.0581***	−0.0581***	−0.0881***	5,640,920
	(0.0063)	(0.0069)	(0.0105)	(0.0141)	(0.0199)	

Notes: Standard Errors in Parentheses. *Pretrend* = Event Times -8 to -7, *Anticipation* = Event Times -5 to -1, *First Year* = Event Times 0 to 3, *Medium Term* = Event Times 4 to 12, *Medium Term* = Event Times 13 and greater; *Complete Sample* = All Observations, *Female* = Only females, *Male* = Only males, *Older Individuals* = Individuals born before or in the median birth year of 1957, *Younger Individuals* = Individuals born after the median birth year of 1957, *Rural* = Individuals living in a county, where the share of inhabitants that live in municipalities with more than 150 Inhabitants per km^2 is less than 75%, *Urban* = Individuals living in a county, where the share of inhabitants that live in municipalities with more than 150 Inhabitants per km^2 is more than 75%, *Comorbidities* = Individuals, that received at least one diagnosis of the Charlson Comorbidity Index by a Physician in the eight quarters before the exit of their GP, *Single Practice* = Leaving physician practiced in a single practice, *Group Practice* = Leaving physician practiced in a group practice.

Table S3: Results: Total GP Costs

	Pretrend	Anticipation	First Year	Medium Term	Long Term	Observations
Complete Sample	0.3334***	−6.4550***	−3.3373***	−0.2760	−0.0739	15,340,080
	(0.1271)	(0.2233)	(0.2501)	(0.2939)	(0.3809)	
Female	0.2894**	−6.6129***	−3.1411***	−0.1382	−0.0856	9,231,320
	(0.1463)	(0.2350)	(0.2594)	(0.3245)	(0.4162)	
Male	0.4040**	−6.2187***	−3.6378***	−0.4885	−0.0642	6,108,760
	(0.1801)	(0.2679)	(0.3314)	(0.3453)	(0.4617)	
Older Individuals	0.5480***	−8.1722***	−3.1031***	0.7634*	0.8463	7,794,800
	(0.1784)	(0.3150)	(0.3564)	(0.4179)	(0.5533)	
Younger Individuals	0.0558	−4.5484***	−3.2812***	−0.9933***	−0.9620***	7,545,280
	(0.1428)	(0.1787)	(0.2138)	(0.2650)	(0.3397)	
Rural	0.1728	−7.3361***	−3.9747***	−0.7013	−0.4287	4,797,560
	(0.2241)	(0.3979)	(0.4590)	(0.4976)	(0.6215)	
Urban	0.4571***	−5.9018***	−2.9037***	0.0846	0.2048	9,839,000
	(0.1447)	(0.2598)	(0.2915)	(0.3529)	(0.4659)	
Comorbidities	0.3504*	−7.9216***	−4.1052***	−0.9743**	−1.3595**	7,874,760
	(0.1922)	(0.3254)	(0.3941)	(0.4505)	(0.5783)	
No Comorbidities	0.3235***	−4.9071***	−2.5194***	0.5068**	1.4429***	7,465,320
	(0.1176)	(0.1667)	(0.1788)	(0.2230)	(0.2953)	
Single Practice	0.5487***	−7.9578***	−3.5082***	0.4810	1.2583***	9,699,160
	(0.1400)	(0.2727)	(0.2852)	(0.3471)	(0.4651)	
Group Practice	−0.0889	−3.7870***	−2.8802***	−1.3060***	−2.0617***	5,640,920
	(0.2297)	(0.2891)	(0.3873)	(0.4607)	(0.6025)	

Notes: Standard Errors in Parentheses. *Pretrend* = Event Times -8 to -7, *Anticipation* = Event Times -5 to -1, *First Year* = Event Times 0 to 3, *Medium Term* = Event Times 4 to 12, *Medium Term* = Event Times 13 and greater; *Complete Sample* = All Observations, *Female* = Only females, *Male* = Only males, *Older Individuals* = Individuals born before or in the median birth year of 1957, *Younger Individuals* = Individuals born after the median birth year of 1957, *Rural* = Individuals living in a county, where the share of inhabitants that live in municipalities with more than 150 Inhabitants per km^2 is less than 75%, *Urban* = Individuals living in a county, where the share of inhabitants that live in municipalities with more than 150 Inhabitants per km^2 is more than 75%, *Comorbidities* = Individuals, that received at least one diagnosis of the Charlson Comorbidity Index by a Physician in the eight quarters before the exit of their GP, *Single Practice* = Leaving physician practiced in a single practice, *Group Practice* = Leaving physician practiced in a group practice.

Table S4: Results: Any Specialist Visit (coefficients multiplied by 100)

	Pretrend	Anticipation	First Year	Medium Term	Long Term	Observations
Complete Sample	−0.0226	0.2309***	0.8842***	0.8809***	0.2015	15,340,080
	(0.0809)	(0.0781)	(0.0972)	(0.1171)	(0.1611)	
Female	−0.0882	0.1680*	0.8841***	0.7833***	0.0600	9,231,320
	(0.1061)	(0.1003)	(0.1207)	(0.1443)	(0.1947)	
Male	0.0792	0.3212***	0.8708***	1.0046***	0.3814*	6,108,760
	(0.1212)	(0.1136)	(0.1410)	(0.1696)	(0.2289)	
Older Individuals	−0.1435	0.2992***	1.3810***	1.4174***	0.5342**	7,794,800
	(0.1091)	(0.1055)	(0.1295)	(0.1545)	(0.2152)	
Younger Individuals	0.1027	0.1610	0.3716***	0.3204**	−0.1729	7,545,280
	(0.1156)	(0.1090)	(0.1326)	(0.1595)	(0.2114)	
Rural	0.1958	0.3808***	1.1587***	1.0845***	0.4899*	4,797,560
	(0.1448)	(0.1439)	(0.1742)	(0.2033)	(0.2821)	
Urban	−0.1708*	0.1307	0.7595***	0.7410***−3e − 04		9,839,000
	(0.0996)	(0.0942)	(0.1184)	(0.1447)	(0.1991)	
Comorbidities	−0.1830*	0.3849***	0.9004***	0.1255	−1.2400***	7,874,760
	(0.1085)	(0.1041)	(0.1291)	(0.1529)	(0.2129)	
No Comorbidities	0.1464	0.0772	0.8877***	1.7106***	1.7543***	7,465,320
	(0.1167)	(0.1107)	(0.1328)	(0.1595)	(0.2087)	
Single Practice	0.0644	0.1673*	0.9657***	1.1544***	0.6793***	9,699,160
	(0.1020)	(0.0983)	(0.1212)	(0.1449)	(0.1916)	
Group Practice	−0.1839	0.3406***	0.7511***	0.4383**	−0.5837**	5,640,920
	(0.1316)	(0.1274)	(0.1571)	(0.1921)	(0.2721)	

Notes: Standard Errors in Parentheses. *Pretrend* = Event Times -8 to -7, *Anticipation* = Event Times -5 to -1, *First Year* = Event Times 0 to 3, *Medium Term* = Event Times 4 to 12, *Medium Term* = Event Times 13 and greater; *Complete Sample* = All Observations, *Female* = Only females, *Male* = Only males, *Older Individuals* = Individuals born before or in the median birth year of 1957, *Younger Individuals* = Individuals born after the median birth year of 1957, *Rural* = Individuals living in a county, where the share of inhabitants that live in municipalities with more than 150 Inhabitants per km^2 is less than 75%, *Urban* = Individuals living in a county, where the share of inhabitants that live in municipalities with more than 150 Inhabitants per km^2 is more than 75%, *Comorbidities* = Individuals, that received at least one diagnosis of the Charlson Comorbidity Index by a Physician in the eight quarters before the exit of their GP, *Single Practice* = Leaving physician practiced in a single practice, *Group Practice* = Leaving physician practiced in a group practice.

Table S5: Results: Number of Specialist Visits

	Pretrend	Anticipation	First Year	Medium Term	Long Term	Observations
Complete Sample	−0.0023	0.0042	0.0459***	0.0563***	0.0376***	15,340,080
	(0.0043)	(0.0041)	(0.0059)	(0.0076)	(0.0103)	
Female	−0.0026	0.0049	0.0520***	0.0610***	0.0356***	9,231,320
	(0.0055)	(0.0055)	(0.0075)	(0.0096)	(0.0128)	
Male	−0.0019	0.0029	0.0359***	0.0479***	0.0385***	6,108,760
	(0.0062)	(0.0058)	(0.0083)	(0.0109)	(0.0148)	
Older Individuals	−0.0036	0.0075	0.0752***	0.0859***	0.0553***	7,794,800
	(0.0063)	(0.0063)	(0.0085)	(0.0107)	(0.0144)	
Younger Individuals	−0.0011	$8e-04$	0.0160**	0.0259***	0.0180	7,545,280
	(0.0055)	(0.0050)	(0.0073)	(0.0096)	(0.0133)	
Rural	0.0000	0.0032	0.0493***	0.0601***	0.0448***	4,797,560
	(0.0071)	(0.0070)	(0.0098)	(0.0122)	(0.0168)	
Urban	−0.0056	0.0021	0.0432***	0.0520***	0.0308**	9,839,000
	(0.0053)	(0.0053)	(0.0076)	(0.0098)	(0.0133)	
Comorbidities	−0.0031	0.0158**	0.0529***	0.0289**	−0.0204	7,874,760
	(0.0066)	(0.0065)	(0.0092)	(0.0118)	(0.0160)	
No Comorbidities	−0.0016	−0.0076	0.0395***	0.0870***	0.1010***	7,465,320
	(0.0048)	(0.0047)	(0.0064)	(0.0080)	(0.0109)	
Single Practice	−0.0009	−0.0030	0.0429***	0.0591***	0.0419***	9,699,160
	(0.0051)	(0.0051)	(0.0071)	(0.0089)	(0.0120)	
Group Practice	−0.0053	0.0170**	0.0516***	0.0528***	0.0315*	5,640,920
	(0.0073)	(0.0070)	(0.0099)	(0.0128)	(0.0176)	

Notes: Standard Errors in Parentheses. *Pretrend* = Event Times -8 to -7, *Anticipation* = Event Times -5 to -1, *First Year* = Event Times 0 to 3, *Medium Term* = Event Times 4 to 12, *Medium Term* = Event Times 13 and greater; *Complete Sample* = All Observations, *Female* = Only females, *Male* = Only males, *Older Individuals* = Individuals born before or in the median birth year of 1957, *Younger Individuals* = Individuals born after the median birth year of 1957, *Rural* = Individuals living in a county, where the share of inhabitants that live in municipalities with more than 150 Inhabitants per km^2 is less than 75%, *Urban* = Individuals living in a county, where the share of inhabitants that live in municipalities with more than 150 Inhabitants per km^2 is more than 75%, *Comorbidities* = Individuals, that received at least one diagnosis of the Charlson Comorbidity Index by a Physician in the eight quarters before the exit of their GP, *Single Practice* = Leaving physician practiced in a single practice, *Group Practice* = Leaving physician practiced in a group practice.

Table S6: Results: Total Specialist Costs

	Pretrend	Anticipation	First Year	Medium Term	Long Term	Observations
Complete Sample	−0.2845	0.3699	1.9123***	2.7115***	1.6677*	15,340,080
	(0.3057)	(0.3329)	(0.4908)	(0.6491)	(0.9143)	
Female	−0.1900	0.4085	2.2997***	3.1762***	1.7565*	9,231,320
	(0.3987)	(0.4222)	(0.5990)	(0.7707)	(1.0669)	
Male	−0.4239	0.2962	1.2896	1.9441*	1.4304	6,108,760
	(0.4690)	(0.5256)	(0.7860)	(1.0914)	(1.4676)	
Older Individuals	−0.4363	0.7284	3.9233***	4.5896***	2.6046*	7,794,800
	(0.5021)	(0.5263)	(0.7362)	(0.9704)	(1.3602)	
Younger Individuals	−0.1822	0.0523	−0.0514	0.8845	0.5165	7,545,280
	(0.3402)	(0.3775)	(0.5704)	(0.7813)	(1.0708)	
Rural	−0.5578	−0.1081	1.4964*	2.8356**	1.8346	4,797,560
	(0.5137)	(0.5613)	(0.8086)	(1.1139)	(1.7386)	
Urban	−0.2532	0.4782	2.0244***	2.5456***	1.5223	9,839,000
	(0.3961)	(0.4261)	(0.6297)	(0.8226)	(1.0966)	
Comorbidities	−0.0887	1.5345***	2.4470***	1.0653	−1.6852	7,874,760
	(0.5138)	(0.5763)	(0.8323)	(1.0989)	(1.5509)	
No Comorbidities	−0.4919*	−0.8416***	1.4031***	4.5675***	5.4905***	7,465,320
	(0.2990)	(0.3090)	(0.4467)	(0.5835)	(0.7929)	
Single Practice	−0.5482	−0.0726	1.3972**	2.5095***	1.3891	9,699,160
	(0.3786)	(0.3985)	(0.5873)	(0.7972)	(1.1171)	
Group Practice	0.1560	1.1370**	2.8406***	3.1366***	2.2852	5,640,920
	(0.5141)	(0.5722)	(0.8240)	(1.0486)	(1.4579)	

Notes: Standard Errors in Parentheses. *Pretrend* = Event Times -8 to -7, *Anticipation* = Event Times -5 to -1, *First Year* = Event Times 0 to 3, *Medium Term* = Event Times 4 to 12, *Medium Term* = Event Times 13 and greater; *Complete Sample* = All Observations, *Female* = Only females, *Male* = Only males, *Older Individuals* = Individuals born before or in the median birth year of 1957, *Younger Individuals* = Individuals born after the median birth year of 1957, *Rural* = Individuals living in a county, where the share of inhabitants that live in municipalities with more than 150 Inhabitants per km^2 is less than 75%, *Urban* = Individuals living in a county, where the share of inhabitants that live in municipalities with more than 150 Inhabitants per km^2 is more than 75%, *Comorbidities* = Individuals, that received at least one diagnosis of the Charlson Comorbidity Index by a Physician in the eight quarters before the exit of their GP, *Single Practice* = Leaving physician practiced in a single practice, *Group Practice* = Leaving physician practiced in a group practice.

Table S7: Results: Any Hospital Visit (coefficients multiplied by 100)

	Pretrend	Anticipation	First Year	Medium Term	Long Term	Observations
Complete Sample	−0.0032	−0.0582**	0.0589*	0.0729*	−0.0020	15,340,080
	(0.0318)	(0.0291)	(0.0340)	(0.0383)	(0.0485)	
Female	0.0072	−0.0684*	0.0787*	0.0553	−0.0261	9,231,320
	(0.0413)	(0.0377)	(0.0438)	(0.0496)	(0.0623)	
Male	−0.0188	−0.0433	0.0275	0.0969*	0.0300	6,108,760
	(0.0494)	(0.0451)	(0.0514)	(0.0583)	(0.0745)	
Older Individuals	−0.0169	−0.0839*	0.1377**	0.1205*	0.0002	7,794,800
	(0.0514)	(0.0468)	(0.0547)	(0.0625)	(0.0785)	
Younger Individuals	0.0085	−0.0302	−0.0174	0.0313	−0.0056	7,545,280
	(0.0357)	(0.0328)	(0.0375)	(0.0416)	(0.0534)	
Rural	−0.0490	−0.0957*	0.0223	0.0382	0.0125	4,797,560
	(0.0576)	(0.0523)	(0.0611)	(0.0695)	(0.0866)	
Urban	0.0362	−0.0406	0.0736*	0.0868*	−0.0190	9,839,000
	(0.0395)	(0.0363)	(0.0416)	(0.0470)	(0.0600)	
Comorbidities	−0.0310	−0.0738	−0.0266	−0.1355**	−0.2934***	7,874,760
	(0.0531)	(0.0483)	(0.0559)	(0.0631)	(0.0792)	
No Comorbidities	0.0255	−0.0405	0.1515***	0.2963***	0.3105***	7,465,320
	(0.0338)	(0.0311)	(0.0358)	(0.0408)	(0.0529)	
Single Practice	0.0066	−0.0299	0.1309***	0.1550***	0.1008*	9,699,160
	(0.0401)	(0.0361)	(0.0429)	(0.0487)	(0.0608)	
Group Practice	−0.0227	−0.1046**	−0.0605	−0.0650	−0.1762**	5,640,920
	(0.0521)	(0.0492)	(0.0551)	(0.0618)	(0.0801)	

Notes: Standard Errors in Parentheses. *Pretrend* = Event Times -8 to -7, *Anticipation* = Event Times -5 to -1, *First Year* = Event Times 0 to 3, *Medium Term* = Event Times 4 to 12, *Medium Term* = Event Times 13 and greater; *Complete Sample* = All Observations, *Female* = Only females, *Male* = Only males, *Older Individuals* = Individuals born before or in the median birth year of 1957, *Younger Individuals* = Individuals born after the median birth year of 1957, *Rural* = Individuals living in a county, where the share of inhabitants that live in municipalities with more than 150 Inhabitants per km^2 is less than 75%, *Urban* = Individuals living in a county, where the share of inhabitants that live in municipalities with more than 150 Inhabitants per km^2 is more than 75%, *Comorbidities* = Individuals, that received at least one diagnosis of the Charlson Comorbidity Index by a Physician in the eight quarters before the exit of their GP, *Single Practice* = Leaving physician practiced in a single practice, *Group Practice* = Leaving physician practiced in a group practice.

Table S8: Results: Any Emergency Visit (coefficients multiplied by 100)

	Pretrend	Anticipation	First Year	Medium Term	Long Term	Observations
Complete Sample	0.0215	−0.0059	0.1089***	0.0601**	0.0327	15,340,080
	(0.0241)	(0.0224)	(0.0253)	(0.0291)	(0.0380)	
Female	0.0044	−0.0172	0.1099***	0.0775**	0.0333	9,231,320
	(0.0307)	(0.0282)	(0.0322)	(0.0370)	(0.0485)	
Male	0.0475	0.0111	0.1069***	0.0323	0.0294	6,108,760
	(0.0389)	(0.0364)	(0.0410)	(0.0465)	(0.0610)	
Older Individuals	0.0314	0.0295	0.2013***	0.1519***	0.1001	7,794,800
	(0.0387)	(0.0359)	(0.0408)	(0.0466)	(0.0613)	
Younger Individuals	0.0062	−0.0337	0.0341	−0.0036	−0.0271	7,545,280
	(0.0295)	(0.0272)	(0.0298)	(0.0335)	(0.0429)	
Rural	0.0661	0.0176	0.1550***	0.1117**	0.1210*	4,797,560
	(0.0448)	(0.0415)	(0.0454)	(0.0529)	(0.0689)	
Urban	0.0059	−0.0224	0.0849***	0.0333	−0.0162	9,839,000
	(0.0294)	(0.0276)	(0.0316)	(0.0357)	(0.0470)	
Comorbidities	0.0147	0.0294	0.0956**	−0.0367	−0.0939	7,874,760
	(0.0399)	(0.0372)	(0.0415)	(0.0474)	(0.0617)	
No Comorbidities	0.0283	−0.0427*	0.1237***	0.1650***	0.1780***	7,465,320
	(0.0272)	(0.0244)	(0.0279)	(0.0317)	(0.0411)	
Single Practice	0.0525*	0.0182	0.1536***	0.1098***	0.0940**	9,699,160
	(0.0294)	(0.0273)	(0.0308)	(0.0360)	(0.0475)	
Group Practice	−0.0327	−0.0477	0.0333	−0.0226	−0.0717	5,640,920
	(0.0419)	(0.0389)	(0.0440)	(0.0483)	(0.0630)	

Notes: Standard Errors in Parentheses. *Pretrend* = Event Times -8 to -7, *Anticipation* = Event Times -5 to -1, *First Year* = Event Times 0 to 3, *Medium Term* = Event Times 4 to 12, *Medium Term* = Event Times 13 and greater; *Complete Sample* = All Observations, *Female* = Only females, *Male* = Only males, *Older Individuals* = Individuals born before or in the median birth year of 1957, *Younger Individuals* = Individuals born after the median birth year of 1957, *Rural* = Individuals living in a county, where the share of inhabitants that live in municipalities with more than 150 Inhabitants per km^2 is less than 75%, *Urban* = Individuals living in a county, where the share of inhabitants that live in municipalities with more than 150 Inhabitants per km^2 is more than 75%, *Comorbidities* = Individuals, that received at least one diagnosis of the Charlson Comorbidity Index by a Physician in the eight quarters before the exit of their GP, *Single Practice* = Leaving physician practiced in a single practice, *Group Practice* = Leaving physician practiced in a group practice.

Table S9: Results: Any Hospitalization with Ambulatory Care Sensitive Condition (coefficients multiplied by 100)

	Pretrend	Anticipation	First Year	Medium Term	Long Term	Observations
Complete Sample	0.0157	0.0153	0.0497***	0.0437***	0.0336**	15,340,080
	(0.0108)	(0.0098)	(0.0113)	(0.0127)	(0.0167)	
Female	0.0131	0.0117	0.0461***	0.0372**	0.0238	9,231,320
	(0.0139)	(0.0126)	(0.0145)	(0.0165)	(0.0213)	
Male	0.0195	0.0207	0.0550***	0.0532***	0.0476*	6,108,760
	(0.0165)	(0.0151)	(0.0173)	(0.0196)	(0.0263)	
Older Individuals	0.0244	0.0304*	0.0974***	0.0917***	0.0583**	7,794,800
	(0.0187)	(0.0170)	(0.0197)	(0.0223)	(0.0295)	
Younger Individuals	0.0049	0.0017	0.0053	0.0017	0.0094	7,545,280
	(0.0099)	(0.0092)	(0.0101)	(0.0113)	(0.0145)	
Rural	0.0265	0.0061	0.0613***	0.0496**	0.0499	4,797,560
	(0.0197)	(0.0177)	(0.0212)	(0.0241)	(0.0308)	
Urban	0.0088	0.0157	0.0434***	0.0418***	0.0283	9,839,000
	(0.0133)	(0.0122)	(0.0138)	(0.0155)	(0.0207)	
Comorbidities	0.0027	0.0207	0.0564***	0.0345	0.0166	7,874,760
	(0.0193)	(0.0176)	(0.0201)	(0.0223)	(0.0291)	
No Comorbidities	0.0291***	0.0097	0.0426***	0.0538***	0.0551***	7,465,320
	(0.0084)	(0.0074)	(0.0090)	(0.0107)	(0.0145)	
Single Practice	0.0186	0.0235**	0.0605***	0.0550***	0.0535**	9,699,160
	(0.0132)	(0.0119)	(0.0141)	(0.0158)	(0.0208)	
Group Practice	0.0105	0.0011	0.0306	0.0231	−0.0016	5,640,920
	(0.0186)	(0.0170)	(0.0188)	(0.0213)	(0.0282)	

Note: Standard Errors in Parentheses. Ambulatory Care Sensitive Conditions include all hospitalizations related to ACSC, as defined by **?**. *Pretrend* = Event Times -8 to -7, *Anticipation* = Event Times -5 to -1, *First Year* = Event Times 0 to 3, *Medium Term* = Event Times 4 and greater; *Complete Sample* = All Observations, *Female* = Only females, *Male* = Only males, *Older Individuals* = Individuals born before or in the median birth year of 1957, *Younger Individuals* = Individuals born after the median birth year of 1957, *Rural* = Individuals living in a county, where the share of inhabitants that live in municipalities with more than 150 Inhabitants per km^2 is less than 75%, *Urban* = Individuals living in a county, where the share of inhabitants that live in municipalities with more than 150 Inhabitants per km^2 is more than 75%, *Comorbidities* = Individuals, that received at least one diagnosis of the Charlson Comorbidity Index by a Physician in the eight quarters before the exit of their GP, *Single Practice* = Leaving physician practiced in a single practice, *Group Practice* = Leaving physician practiced in a group practice.

Table S10: Results: New Diagnoses (coefficients multiplied by 100)

	Pretrend	Anticipation	First Year	Medium Term	Long Term	Observations
MI	−0.0077	−0.0091	0.0156**	−0.0060	−0.0116	14,956,578
	(0.0079)	(0.0069)	(0.0076)	(0.0081)	(0.0102)	
CHF	0.0016	−0.0018	0.0737***	0.0033	−0.0309	14,956,578
	(0.0141)	(0.0132)	(0.0144)	(0.0163)	(0.0223)	
PDV	−0.0136	−0.0015	0.1260***	0.0698***	0.0407*	14,956,578
	(0.0168)	(0.0142)	(0.0158)	(0.0179)	(0.0241)	
CEVD	−0.0245	−0.0144	0.0722***	0.0224	−0.0081	14,956,578
	(0.0173)	(0.0156)	(0.0174)	(0.0191)	(0.0241)	
Dementia	0.0099*	0.0079	0.0190***	0.0088	−0.0097	14,956,578
	(0.0058)	(0.0056)	(0.0066)	(0.0074)	(0.0097)	
CPD	0.0105	−0.0142	−0.0053	−0.0856**	−0.1815***	14,956,578
	(0.0311)	(0.0264)	(0.0298)	(0.0350)	(0.0485)	
Rehuma	−0.0045	−0.0112	−0.0037	−0.0271**	−0.0420***	14,956,578
	(0.0105)	(0.0094)	(0.0098)	(0.0112)	(0.0150)	
PUD	−0.0041	−0.0083	−0.0023	−0.0108	−0.0172*	14,956,578
	(0.0065)	(0.0056)	(0.0061)	(0.0069)	(0.0089)	
MLD	0.0046	−0.0117	0.1358***	0.0438*	−0.0364	14,956,578
	(0.0240)	(0.0193)	(0.0215)	(0.0255)	(0.0360)	
Diabetes	−0.0186	−0.0383**	0.0318	−0.0687***	−0.1337***	14,956,578
	(0.0223)	(0.0188)	(0.0202)	(0.0235)	(0.0315)	
Diab. w. Complications	−0.0066	0.0030	0.0773***	0.0478***	0.0221	14,956,578
	(0.0136)	(0.0122)	(0.0133)	(0.0161)	(0.0219)	
HP	0.0042	−0.0015	0.0153***	−0.0013	−0.0067	14,956,578
	(0.0058)	(0.0051)	(0.0057)	(0.0063)	(0.0083)	
Renal Disease	−0.0042	−0.0016	0.1331***	0.0628***	0.0284	14,956,578
	(0.0121)	(0.0115)	(0.0146)	(0.0164)	(0.0225)	
Cancer	0.0029	−0.0224*	0.0040	−0.0460***	−0.0741***	14,956,578
	(0.0152)	(0.0132)	(0.0145)	(0.0158)	(0.0204)	
MSLD	−0.0011	−0.0020	−0.0017	−0.0037	−0.0033	14,956,578
	(0.0025)	(0.0023)	(0.0026)	(0.0029)	(0.0035)	
Met. Cancer	0.0027	0.0041	0.0094**	0.0046	0.0017	14,956,578
	(0.0046)	(0.0042)	(0.0047)	(0.0054)	(0.0073)	
AIDS	−0.0042	−0.0042	−0.0048	−0.0056	−0.0064	14,956,578
	(0.0057)	(0.0052)	(0.0053)	(0.0056)	(0.0062)	

Note: Standard Errors in Parentheses. *Pretrend* = Event Times -8 to -7, *Anticipation* = Event Times -5 to -1, *First Year* = Event Times 0 to 3, *Medium Term* = Event Times 4 to 12, *Medium Term* = Event Times 13 and greater; All estimations based on the complete sample of treated individuals. The outcome variable is equal to one in the first quarter in the observational period that respective disease (as defined by the Chalson Comorbidity index (**?**)) is diagnosed and 0 else.

Table S11: Results: Tests (coefficients multiplied by 100)

	Pretrend	Anticipation	First Year	Medium Term	Long Term	Observations
Blood Counts	0.0310	0.0137	−0.1371**	−0.3148***	−0.3299***	15,34,080
	(0.0271)	(0.0287)	(0.0597)	(0.0830)	(0.0995)	
Total Protein	0.0083	0.0023	−0.0628*	−0.1729***	−0.1555***	15,340,080
	(0.0161)	(0.0177)	(0.0355)	(0.0458)	(0.0527)	
Beta Blockers	−0.0187	−0.0489	−0.0881	−0.1125	−0.2125**	15,340,080
	(0.0431)	(0.0418)	(0.0545)	(0.0717)	(0.0963)	
ACE Inhibitors	0.0548	0.0386	0.4024***	0.6941***	0.6514***	15,340,080
	(0.0369)	(0.0357)	(0.0493)	(0.0657)	(0.0891)	
Antibiotics	0.0098	−0.0172	−0.1574***	−0.1761***	−0.1905***	15,340,080
	(0.0232)	(0.0203)	(0.0250)	(0.0288)	(0.0369)	

Note: Standard Errors in Parentheses. *Pretrend* = Event Times -8 to -7, *Anticipation* = Event Times -5 to -1, *First Year* = Event Times 0 to 3, *Medium Term* = Event Times 4 to 12, *Medium Term* = Event Times 13 and greater; All estimations based on the complete sample of treated individuals. Complete Blood Count as defined by EBM No.32122. Total Protein as defined by EBM No.32056. ACE Inhibitors include all prescriptions with ATC C09a and C09b. Beta Blockers include all prescriptions with ATC C07. Antibiotics include all prescriptions with ATC J01.

Table S12: Additional effects by specific sub-groups (coefficients multiplied by 100)

	Any Emergency visit		Any ACSC visit		
	First Year	Med. run	First Year	Med. run	Observations
Complete Sample:	0.1089	0.0601	0.0497	0.0437	15,340,080
	(0.0253)	(0.0291)	(0.0113)	(0.0127)	
	[0.0000]	[0.0526]	[0.0000]	[0.0009]	
Gender:					
– Female	0.1099	0.0775	0.0461	0.0372	9,231,320
	(0.0322)	(0.0370)	(0.0145)	(0.0165)	
	[0.0011]	[0.0516]	[0.0025]	[0.0357]	
– Male	0.1069	0.0323	0.0550	0.0532	6,108,760
	(0.0410)	(0.0465)	(0.0173)	(0.0196)	
	[0.0173]	[0.5342]	[0.0034]	[0.0134]	
Individuals:					
– Older	0.2013	0.1519	0.0974	0.0917	7,794,800
	(0.0408)	(0.0466)	(0.0197)	(0.0223)	
	[0.0000]	[0.0018]	[0.0000]	[0.0001]	
– Younger	0.0341	−0.0036	0.0053	0.0017	7,545,280
	(0.0298)	(0.0335)	(0.0101)	(0.0113)	
	[0.3369]	[0.9282]	[0.7050]	[0.9282]	
Area:					
– Rural	0.1550	0.1117	0.0613	0.0496	4,797,560
	(0.0454)	(0.0529)	(0.0212)	(0.0241)	
	[0.0015]	[0.0515]	[0.0081]	[0.0555]	
– Urban	−0.0849	0.0333	0.0434	0.0418	9,839,000
	(0.0316)	(0.0357)	(0.0138)	(0.0155)	
	[0.0122]	[0.3863]	[0.0039]	[0.0122]	
Comorbidities:					
– with	0.0945	−0.0112	0.0553	0.0351	8,952,560
	(0.0381)	(0.0436)	(0.0181)	(0.0202)	
	[0.0196]	[0.8210]	[0.0035]	[0.1041]	
– without	0.1127	0.1384	0.0353	0.0456	6,387,520
	(0.0294)	(0.0336)	(0.0095)	(0.0111)	
	[0.0002]	[0.0001]	[0.0003]	[0.0001]	
Practice:					
– Single	0.1536	0.1098	0.0605	0.0550	9,699,160
	(0.0308)	(0.0360)	(0.0141)	(0.0158)	
	[0.0000]	[0.0039]	[0.0000]	[0.0010]	
– Group	0.0333	−0.0226	0.0306	0.0231	5,640,920
	(0.0440)	(0.0483)	(0.0188)	(0.0213)	
	[0.5236]	[0.7011]	[0.1604]	[0.3508]	

Notes: Standard Errors in Parentheses. Adjusted p-values following **?** with m = 34 (the number of presented point estimates per sub-sample), applied for each sub-sample separately, in brackets. *First Year* = Event Times 0 to 3, *Medium Term* = Event Times 4 to 12; *Complete Sample* = All Observations, *Female* = Only females, *Male* = Only males, *Older Individuals* = Individuals born before or in the median birth year of 1957, *Younger Individuals* = Individuals born after the median birth year of 1957, *Rural* = Individuals living in a county, where the share of inhabitants that live in municipalities with more than 150 Inhabitants per km^2 is less than 75%, *Urban* = Individuals living in a county, where the share of inhabitants that live in municipalities with more than 150 Inhabitants per km^2 is more than 75%, *Comorbidities* = Individuals, that received at least one diagnosis of the Charlson Comorbidity Index by a Physician in the eight quarters before the exit of their GP, *Single Practice* = Leaving physician practiced in a single practice, *Group Practice* = Leaving physician practiced in a group practice.

Table S13: Additional effects by specific sub-groups

	Number of GP visit		Number of specialist visit		
	First Year	Med. run	First Year	Med. run	Observations
Complete Sample:	−0.0515	−0.0388	0.0459	0.0563	15,340,080
	(0.0068)	(0.0091)	(0.0059)	(0.0076)	
	[0.0000]	[0.0000]	[0.0000]	[0.0000]	
Gender:					
– Female	−0.0443	−0.0373	0.0520	0.0610	9,231,320
	(0.0074)	(0.0101)	(0.0075)	(0.0096)	
	[0.0000]	[0.0004]	[0.0000]	[0.0000]	
– Male	−0.0627	−0.0418	0.0359	0.0479	6,108,760
	(0.0083)	(0.0108)	(0.0083)	(0.0109)	
	[0.0000]	[0.0003]	[0.0000]	[0.0000]	
Individuals:					
– Older	−0.0175	−0.0170	0.0752	0.0859	7,794,800
	(0.0092)	(0.0124)	(0.0085)	(0.0107)	
	[0.0828]	[0.1941]	[0.0000]	[0.0000]	
– Younger	−0.0837	−0.0597	0.0160	0.0259	7,545,280
	(0.0070)	(0.0091)	(0.0073)	(0.0096)	
	[0.0000]	[0.0000]	[0.0584]	[0.0173]	
Area:					
– Rural	−0.0686	−0.0632	0.0493	0.0601	4,797,560
	(0.0112)	(0.0145)	(0.0098)	(0.0122)	
	[0.0000]	[0.0000]	[0.0000]	[0.0000]	
– Urban	−0.0451	−0.0300	0.0432	0.0520	9,839,000
	(0.0084)	(0.0115)	(0.0076)	(0.0098)	
	[0.0000]	[0.0145]	[0.0000]	[0.0000]	
Comorbidities:					
– with	−0.0533	−0.0558	0.0594	0.0509	8,952,560
	(0.0091)	(0.0120)	(0.0085)	(0.0110)	
	[0.0000]	[0.0000]	[0.0000]	[0.0000]	
– without	−0.0420	0.0022	0.0361	0.0788	6,387,520
	(0.0061)	(0.0083)	(0.0066)	(0.0083)	
	[0.0000]	[0.8119]	[0.0000]	[0.0000]	
Practice:					
– Single	−0.0475	−0.0267	0.0429	0.0591	9,699,160
	(0.0082)	(0.0110)	(0.0071)	(0.0089)	
	[0.0000]	[0.0208]	[0.0000]	[0.0000]	
– Group	−0.0581	−0.0581	0.0516	0.0528	5,640,920
	(0.0105)	(0.0141)	(0.0099)	(0.0128)	
	[0.0000]	[0.0001]	[0.0000]	[0.0001]	

Notes: Standard Errors in Parentheses. Adjusted p-values following **?** with m = 34 (the number of presented point estimates per sub-sample), applied for each sub-sample separately, in brackets. *First Year* = Event Times 0 to 3, *Medium Term* = Event Times 4 to 12; *Complete Sample* = All Observations, *Female* = Only females, *Male* = Only males, *Older Individuals* = Individuals born before or in the median birth year of 1957, *Younger Individuals* = Individuals born after the median birth year of 1957, *Rural* = Individuals living in a county, where the share of inhabitants that live in municipalities with more than 150 Inhabitants per km^2 is less than 75%, *Urban* = Individuals living in a county, where the share of inhabitants that live in municipalities with more than 150 Inhabitants per km^2 is more than 75%, *Comorbidities* = Individuals, that received at least one diagnosis of the Charlson Comorbidity Index by a Physician in the eight quarters before the exit of their GP, *Single Practice* = Leaving physician practiced in a single practice, *Group Practice* = Leaving physician practiced in a group practice.

Table S14: Additional effects by specific sub-groups

	GP Costs		Specialist costs		
	First Year	Med. run	First Year	Med. run	Observations
Complete Sample:	−3.3373	−0.2760	1.9123	2.7115	15,340,080
	(0.2501)	(0.2939)	(0.4908)	(0.6491)	
	[0.0000]	[0.3694]	[0.0002]	[0.0001]	
Gender:					
– Female	−3.1411	−0.1382	2.2997	3.1762	9,231,320
	(0.2594)	(0.3245)	(0.5990)	(0.7707)	
	[0.0000]	[0.7120]	[0.0003]	[0.0001]	
– Male	−3.6378	−0.4885	1.2896	1.9441	6,108,760
	(0.3314)	(0.3453)	(0.7860)	(1.0914)	
	[0.0000]	[0.2055]	[0.1430]	[0.1158]	
Individuals:					
– Older	−3.1031	0.7634	3.9233	4.5896	7,794,800
	(0.3564)	(0.4179)	(0.7362)	(0.9704)	
	[0.0000]	[0.0921]	[0.0000]	[0.0000]	
– Younger	−3.2812	−0.9933	−0.0514	0.8845	7,545,280
	(0.2138)	(0.2650)	(0.5704)	(0.7813)	
	[0.0000]	[0.0007]	[0.9282]	[0.3369]	
Area:					
– Rural	−3.9747	−0.7013	1.4964	2.8356	4,797,560
	(0.4590)	(0.4976)	(0.8086)	(1.1139)	
	[0.0000]	[0.1929]	[0.0874]	[0.0186]	
– Urban	−2.9037	0.0846	2.0244	2.5456	9,839,000
	(0.2915)	(0.3529)	(0.6297)	(0.8226)	
	[0.0000]	[0.8106]	[0.0034]	[0.0042]	
Comorbidities:					
– with	−3.9436	−0.6724	2.6316	2.2076	8,952,560
	(0.3587)	(0.4159)	(0.7608)	(1.0068)	
	[0.0000]	[0.1287]	[0.0009]	[0.0386]	
– without	−2.7740	0.0775	1.0598	3.8386	6,387,520
	(0.1819)	(0.2262)	(0.4688)	(0.6100)	
	[0.0000]	[0.7778]	[0.0289]	[0.0000]	
Practice:					
– Single	−3.5082	0.4810	1.3972	2.5095	9,699,160
	(0.2852)	(0.3471)	(0.5873)	(0.7972)	
	[0.0000]	[0.1944]	[0.0227]	[0.0031]	
– Group	−2.8802	−1.3060	2.8406	3.1366	5,640,920
	(0.3873)	(0.4607)	(0.8240)	(1.0486)	
	[0.0000]	[0.0104]	[0.0018]	[0.0068]	

Notes: Standard Errors in Parentheses. Adjusted p-values following **?** with m = 34 (the number of presented point estimates per sub-sample), applied for each sub-sample separately, in brackets. *First Year* = Event Times 0 to 3, *Medium Term* = Event Times 4 to 12; *Complete Sample* = All Observations, *Female* = Only females, *Male* = Only males, *Older Individuals* = Individuals born before or in the median birth year of 1957, *Younger Individuals* = Individuals born after the median birth year of 1957, *Rural* = Individuals living in a county, where the share of inhabitants that live in municipalities with more than 150 Inhabitants per km^2 is less than 75%, *Urban* = Individuals living in a county, where the share of inhabitants that live in municipalities with more than 150 Inhabitants per km^2 is more than 75%, *Comorbidities* = Individuals, that received at least one diagnosis of the Charlson Comorbidity Index by a Physician in the eight quarters before the exit of their GP, *Single Practice* = Leaving physician practiced in a single practice, *Group Practice* = Leaving physician practiced in a group practice.

Table S15: Additional effects by specific sub-groups (coefficients multiplied by 100)

	CHF Diagnoses		CPD Diagnoses		Diabetes Diagnoses		
	First Year	Med. run	First Year	Med. run	First Year	Med. run	Observations
Complete Sample:	0.0737	0.0033	−0.0053	−0.0856	0.0318	−0.0687	14,956,578
	(0.0144)	(0.0163)	(0.0298)	(0.0350)	(0.0202)	(0.0235)	
	[0.0000]	[0.8589]	[0.8589]	[0.0216]	[0.1281]	[0.0054]	
Gender:							
– Female	−0.0004	−0.0015	−0.0825	0.0327	−0.0653	900.0537	
	(0.0165)	(0.0186)	(0.0351)	(0.0405)	(0.0217)	(0.0249)	
	[0.0003]	[0.9948]	[0.9948]	[0.0569]	[0.1602]	[0.0134]	
– Male	0.0902	0.0085	−0.0111	−0.0909	0.0302	−0.0736	5,956,041
	(0.0203)	(0.0225)	(0.0413)	(0.0466)	(0.0291)	(0.0336)	
	[0.0000]	[0.7271]	[0.7890]	[0.0828]	[0.3517]	[0.0482]	
Individuals:							
– Older	0.1470	0.0203	0.0534	−0.0242	0.1149	−0.0526	7,599,930
	(0.0257)	(0.0289)	(0.0372)	(0.0425)	(0.0314)	(0.0361)	
	[0.0000]	[0.5304]	[0.1776]	[0.6042]	[0.0004]	[0.1762]	
– Younger	0.0036	−0.0469	−0.1281	−0.0119	−0.0304 7,356,648		
	(0.0086)	(0.0096)	(0.0384)	(0.0445)	(0.0156)	(0.0180)	
	[0.2308]	[0.7749]	[0.3142]	[0.0124]	[0.5484]	[0.1429]	
Area:							
– Rural	0.1695	0.0690	−0.0704	−0.1481	0.0888	−0.0124	4,677,621
	(0.0255)	(0.0282)	(0.0469)	(0.0551)	(0.0334)	(0.0375)	
	[0.0000]	[0.0226]	[0.1681]	[0.0144]	[0.0149]	[0.7622]	
– Urban	0.0239	−0.0328	0.0265	−0.0653	0.0108	−0.0918	9,593,025
	(0.0179)	(0.0207)	(0.0384)	(0.0448)	(0.0251)	(0.0295)	
	[0.2130]	[0.1415]	[0.5202]	[0.1764]	[0.6883]	[0.0042]	
Comorbidities:							
– with	0.0140	−0.1180	−0.9285	−1.2854	−0.1842	−0.4261	8,728,746
	(0.0235)	(0.0269)	(0.0467)	(0.0528)	(0.0336)	(0.0387)	
	[0.6042]	[0.0000]	[0.0000]	[0.0000]	[0.0000]	[0.0000]	
– without	0.1236	0.1208	1.0772	1.1952	0.1961	0.2167	6,227,832
	(0.0070)	(0.0077)	(0.0182)	(0.0206)	(0.0071)	(0.0087)	
	[0.0000]	[0.0000]	[0.0000]	[0.0000]	[0.0000]	[0.0000]	
Practice:							
– Single	0.1114	0.0251	0.0312	−0.0474	0.0499	−0.0760	9,456,681
	(0.0189)	(0.0209)	(0.0387)	(0.0456)	(0.0257)	(0.0292)	
	[0.0000]	[0.2498]	[0.4191]	[0.3082]	[0.0655]	[0.0146]	
– Group	0.0074	−0.0359	−0.0684	−0.1517	−0.0013	−0.0594	5,499,897
	(0.0208)	(0.0245)	(0.0426)	(0.0489)	(0.0303)	(0.0360)	
	[0.7674]	[0.2030]	[0.1604]	[0.0050]	[0.9651]	[0.1593]	

Notes: Standard Errors in Parentheses. Adjusted p-values following **?** with m = 34 (the number of presented point estimates per sub-sample), applied for each sub-sample separately, in brackets. *First Year* = Event Times 0 to 3, *Medium Term* = Event Times 4 to 12; *Complete Sample* = All Observations, *Female* = Only females, *Male* = Only males, *Older Individuals* = Individuals born before or in the median birth year of 1957, *Younger Individuals* = Individuals born after the median birth year of 1957, *Rural* = Individuals living in a county, where the share of inhabitants that live in municipalities with more than 150 Inhabitants per km^2 is less than 75%, *Urban* = Individuals living in a county, where the share of inhabitants that live in municipalities with more than 150 Inhabitants per km^2 is more than 75%, *Comorbidities* = Individuals, that received at least one diagnosis of the Charlson Comorbidity Index by a Physician in the eight quarters before the exit of their GP, *Single Practice* = Leaving physician practiced in a single practice, *Group Practice* = Leaving physician practiced in a group practice.

Table S16: Additional effects by specific sub-groups (coefficients multiplied by 100)

	Complete Blood Count		Total Protein		
	First Year	Med. run	First Year	Med. run	Observations
Complete Sample:	−0.1371	−0.3148	−0.0628	−0.1729	15,340,080
	(0.0597)	(0.0830)	(0.0355)	(0.0458)	
	[0.0307]	[0.0003]	[0.0966]	[0.0003]	
Gender:					
– Female	−0.1749	−0.3723	−0.0690	−0.2103	9,231,320
	(0.0662)	(0.0915)	(0.0395)	(0.0499)	
	[0.0133]	[0.0001]	[0.1017]	[0.0001]	
– Male	−0.0798	−0.2276	−0.0531	−0.1160	6,108,760
	(0.0604)	(0.0817)	(0.0375)	(0.0479)	
	[0.2349]	[0.0114]	[0.2055]	[0.0276]	
Individuals:					
– Older	−0.1451	−0.4322	−0.0791	−0.2663	7,794,800
	(0.0828)	(0.1144)	(0.0515)	(0.0656)	
	[0.1044]	[0.0003]	[0.1569]	[0.0001]	
– Younger	−0.1323	−0.2011	−0.0459	−0.0804	7,545,280
	(0.0476)	(0.0639)	(0.0257)	(0.0332)	
	[0.0143]	[0.0056]	[0.1209]	[0.0330]	
Area:					
– Rural	−0.1105	−0.2460	−0.0653	−0.1620	4,797,560
	(0.0734)	(0.0949)	(0.0520)	(0.0646)	
	[0.1681]	[0.0170]	[0.2450]	[0.0197]	
– Urban	−0.1439	−0.3256	−0.0588	−0.1809	9,839,000
	(0.0784)	(0.1095)	(0.0468)	(0.0609)	
	[0.0945]	[0.0056]	[0.2371]	[0.0056]	
Comorbidities:					
– with	−0.1821	−0.4476	−0.0963	−0.2608	8,952,560
	(0.0815)	(0.1128)	(0.0501)	(0.0639)	
	[0.0362]	[0.0001]	[0.0715]	[0.0001]	
– without	−0.0524	−0.0875	−0.0054	−0.0233	6,387,520
	(0.0418)	(0.0549)	(0.0228)	(0.0293)	
	[0.2379]	[0.1303]	[0.8119]	[0.4691]	
Practice:					
– Single	−0.0926	−0.2717	−0.0445	−0.1385	9,699,160
	(0.0743)	(0.1053)	(0.0375)	(0.0476)	
	[0.2411]	[0.0146]	[0.2498]	[0.0059]	
– Group	−0.2160	−0.3873	−0.0876	−0.2203	5,640,920
	(0.0855)	(0.1145)	(0.0632)	(0.0821)	
	[0.0215]	[0.0021]	[0.2257]	[0.0146]	

Notes: Standard Errors in Parentheses. Adjusted p-values following **?** with m = 34 (the number of presented point estimates per sub-sample), applied for each sub-sample separately, in brackets. *First Year* = Event Times 0 to 3, *Medium Term* = Event Times 4 to 12; *Complete Sample* = All Observations, *Female* = Only females, *Male* = Only males, *Older Individuals* = Individuals born before or in the median birth year of 1957, *Younger Individuals* = Individuals born after the median birth year of 1957, *Rural* = Individuals living in a county, where the share of inhabitants that live in municipalities with more than 150 Inhabitants per km^2 is less than 75%, *Urban* = Individuals living in a county, where the share of inhabitants that live in municipalities with more than 150 Inhabitants per km^2 is more than 75%, *Comorbidities* = Individuals, that received at least one diagnosis of the Charlson Comorbidity Index by a Physician in the eight quarters before the exit of their GP, *Single Practice* = Leaving physician practiced in a single practice, *Group Practice* = Leaving physician practiced in a group practice.

Table S17: Additional effects by specific sub-groups (coefficients multiplied by 100)

	ACE Inh. Prescriptions		Beta Blocker Prescr.		Antibiotics Prescr.		
	First Year	Med. run	First Year	Med. run	First Year	Med. run	Observations
Complete Sample:	0.4024	0.6941	−0.0881	−0.1125	−0.1574	−0.1761	15,340,080
	(0.0493)	(0.0657)	(0.0545)	(0.0717)	(0.0250)	(0.0288)	
	[0.0000]	[0.0000]	[0.1243]	[0.1281]	[0.0000]	[0.0000]	
Gender:							
– Female	0.3853	0.6458	−0.1004	−0.1076	−0.1529	−0.1608	9,231,320
	(0.0596)	(0.0779)	(0.0685)	(0.0905)	(0.0301)	(0.0344)	
	[0.0000]	[0.0000]	[0.1673]	[0.2657]	[0.0000]	[0.0000]	
– Male	0.4244	0.7614	−0.0710	−0.1217	−0.1641	−0.1995	6,108,760
	(0.0800)	(0.1050)	(0.0802)	(0.1031)	(0.0338)	(0.0381)	
	[0.0000]	[0.0000]	[0.4265]	[0.2889]	[0.0000]	[0.0000]	
Individuals:							
– Older	0.6823	1.0126	−0.0022	0.0540	−0.1324	−0.1520	7,794,800
	(0.0839)	(0.1099)	(0.0920)	(0.1199)	(0.0305)	(0.0348)	
	[0.0000]	[0.0000]	[0.9806]	[0.6725]	[0.0000]	[0.0000]	
– Younger	0.1197	0.3780	−0.0933	−0.1311	−0.1809	−0.1993	7,545,280
	(0.0454)	(0.0631)	(0.0492)	(0.0645)	(0.0326)	(0.0372)	
	[0.0190]	[0.0000]	[0.0983]	[0.0797]	[0.0000]	[0.0000]	
Area:							
– Rural	0.3883	0.6550	−0.0102	0.0443	−0.2204	−0.2388	4,797,560
	(0.0860)	(0.1184)	(0.1005)	(0.1272)	(0.0455)	(0.0525)	
	[0.0000]	[0.0000]	[0.9190]	[0.7622]	[0.0000]	[0.0000]	
– Urban	0.4091	0.6912	−0.1167	−0.1836	−0.1337	−0.1609	9,839,000
	(0.0616)	(0.0813)	(0.0662)	(0.0890)	(0.0307)	(0.0354)	
	[0.0000]	[0.0000]	[0.1018]	[0.0603]	[0.0000]	[0.0000]	
Comorbidities:							
– with	0.4776	0.6698	0.0133	−0.1268	−0.2122	−0.2321	8,952,560
	(0.0718)	(0.0952)	(0.0802)	(0.1039)	(0.0348)	(0.0402)	
	[0.0000]	[0.0000]	[0.8680]	[0.2611]	[0.0000]	[0.0000]	
– without	0.3153	0.7241	−0.3304	−0.2994	−0.0688	−0.0889	6,387,520
	(0.0567)	(0.0764)	(0.0609)	(0.0815)	(0.0288)	(0.0320)	
	[0.0000]	[0.0000]	[0.0000]	[0.0003]	[0.0214]	[0.0072]	
Practice:							
– Single	0.3819	0.6713	−0.1726	−0.1638	−0.1959	−0.2123	9,699,160
	(0.0607)	(0.0822)	(0.0682)	(0.0902)	(0.0322)	(0.0368)	
	[0.0000]	[0.0000]	[0.0163]	[0.0843]	[0.0000]	[0.0000]	
– Group	0.4386	0.7297	0.0655	−0.0134	−0.0920	−0.1156	5,640,920
	(0.0831)	(0.1062)	(0.0890)	(0.1143)	(0.0366)	(0.0425)	
	[0.0000]	[0.0000]	[0.5236]	[0.9338]	[0.0215]	[0.0138]	

Notes: Standard Errors in Parentheses. Adjusted p-values following **?** with m = 34 (the number of presented point estimates per sub-sample), applied for each sub-sample separately, in brackets. *First Year* = Event Times 0 to 3, *Medium Term* = Event Times 4 to 12; *Complete Sample* = All Observations, *Female* = Only females, *Male* = Only males, *Older Individuals* = Individuals born before or in the median birth year of 1957, *Younger Individuals* = Individuals born after the median birth year of 1957, *Rural* = Individuals living in a county, where the share of inhabitants that live in municipalities with more than 150 Inhabitants per km^2 is less than 75%, *Urban* = Individuals living in a county, where the share of inhabitants that live in municipalities with more than 150 Inhabitants per km^2 is more than 75%, *Comorbidities* = Individuals, that received at least one diagnosis of the Charlson Comorbidity Index by a Physician in the eight quarters before the exit of their GP, *Single Practice* = Leaving physician practiced in a single practice, *Group Practice* = Leaving physician practiced in a group practice.

Table S18: Effects by absorbing GP's practice characteristics – ambulatory care utilization I, diagnoses, and prescriptions (coefficients multiplied by 100)

	First Year	Med. run	First Year	Med. run	First Year	Med. run	Observations
	Any GP visit		Any specialist visit		Any hospital visit		
Switch to:							
– Single	−2.4136	−2.4889	1.0024	0.9809	0.0920	0.1299	8,332,360
	(0.1402)	(0.1677)	(0.1263)	(0.1517)	(0.0460)	(0.0521)	
	[0.0000]	[0.0000]	[0.0000]	[0.0000]	[0.0675]	[0.0205]	
– Group	−3.3340	−2.8603	1.0813	0.9891	0.0387	0.0138	6,641,920
	(0.1648)	(0.1940)	(0.1483)	(0.1813)	(0.0519)	(0.0586)	
	[0.0000]	[0.0000]	[0.0000]	[0.0000]	[0.5167]	[0.8392]	
– Larger	−2.8386	−2.3560	1.0262	0.9068	0.1128	0.1423	8,858,040
	(0.1419)	(0.1681)	(0.1236)	(0.1479)	(0.0442)	(0.0497)	
	[0.0000]	[0.0000]	[0.0000]	[0.0000]	[0.0166]	[0.0071]	
– Smaller	−1.5142	−1.5395	1.3858	1.5732	0.0426	0.0241	5,505,240
	(0.1722)	(0.2093)	(0.1602)	(0.1946)	(0.0583)	(0.0664)	
	[0.0000]	[0.0000]	[0.0000]	[0.0000]	[0.5269]	[0.7165]	

	CHF Diagnoses		CPD Diagnoses		Diabetes Diagnoses		
Switch to:							
– Single	0.0953	0.0180	0.0502	−0.0279	0.0395	−0.0594	8,124,051
	(0.0201)	(0.0224)	(0.0396)	(0.0464)	(0.0276)	(0.0317)	
	[0.0000]	[0.4777]	[0.2397]	[0.6009]	[0.1844]	[0.0797]	
– Group	0.0582	−0.0034	−0.0433	−0.1247	0.0535	−0.0436	6,475,872
	(0.0191)	(0.0220)	(0.0411)	(0.0468)	(0.0266)	(0.0311)	
	[0.0045]	[0.8769]	[0.3429]	[0.0132]	[0.0685]	[0.2018]	
– Larger	0.1126	0.0178	0.0443	−0.0271	0.0851	−0.0301	8,636,589
	(0.0174)	(0.0192)	(0.0385)	(0.0436)	(0.0273)	(0.0308)	
	[0.0000]	[0.3893]	[0.3042]	[0.5681]	[0.0033]	[0.3717]	
– Smaller	0.0522	0.0138	0.0177	−0.0588	0.0205	−0.0386	5,367,609
	(0.0260)	(0.0299)	(0.0469)	(0.0558)	(0.0318)	(0.0382)	
	[0.0695]	[0.6860]	[0.7165]	[0.3823]	[0.5701]	[0.3893]	

	ACE Inh. Prescriptions		Beta Blocker Prescr.		Antibiotics Prescr.		
Switch to:							
– Single	0.3212	0.5564	−0.0154	−0.0527	−0.1418	−0.1774	8,332,360
	(0.0675)	(0.0910)	(0.0751)	(0.0988)	(0.0330)	(0.0380)	
	[0.0000]	[0.0000]	[0.8633]	[0.6310]	[0.0000]	[0.0000]	
– Group	0.5351	0.8986	−0.1389	−0.1291	−0.1693	−0.1729	6,641,920
	(0.0739)	(0.0962)	(0.0810)	(0.1061)	(0.0356)	(0.0406)	
	[0.0000]	[0.0000]	[0.1226]	[0.2715]	[0.0000]	[0.0001]	
– Larger	0.3795	0.6776	−0.1376	−0.1568	−0.1419	−0.1440	8,858,040
	(0.0662)	(0.0881)	(0.0716)	(0.0937)	(0.0305)	(0.0338)	
	[0.0000]	[0.0000]	[0.0743]	[0.1230]	[0.0000]	[0.0000]	
– Smaller	0.5724	0.8960	0.1051	0.1470	−0.1891	−0.2267	5,505,240
	(0.0841)	(0.1117)	(0.0936)	(0.1232)	(0.0443)	(0.0516)	
	[0.0000]	[0.0000]	[0.3557]	[0.3301]	[0.0001]	[0.0000]	

Notes: Standard Errors in Parentheses. Adjusted p-values following **?** with m = 34 (the number of presented point estimates per sub-sample), applied for each sub-sample separately, in brackets. *First Year* = Event Times 0 to 3, *Medium Term* = Event Times 4 to 12; *Switch to Single* = first practice a patient visits after the exit is a single practice. *Switch to Group* = first practice a patient visits after the exit is a group practice. *Switch to Larger* = first GP a patient visits after the exit has more patients than practice of leaving GP. *Switch to Smaller* = first GP a patient visits after the exit has less patients than practice of leaving GP.

Table S19: Effects by absorbing GP's practice characteristics – hospitalizations and diagnostic tests (coefficients multiplied by 100)

	First Year	Med. run	First Year	Med. run	Observations
	Any Emergency visit		Any ACSC visit		
Switch to:					
– Single	0.1302	0.0758	0.0442	0.0337	8,332,360
	(0.0342)	(0.0397)	(0.0156)	(0.0175)	
	[0.0003]	[0.0763]	[0.0090]	[0.0763]	
– Group	0.1101	0.0664	0.0603	0.0594	6,641,920
	(0.0387)	(0.0437)	(0.0167)	(0.0191)	
	[0.0080]	[0.1683]	[0.0009]	[0.0039]	
Switch to:					
– Larger	0.0943	0.0529	0.0495	0.0390	8,858,040
	(0.0331)	(0.0385)	(0.0147)	(0.0169)	
	[0.0072]	[0.2136]	[0.0017]	[0.0295]	
– Smaller	0.2089	0.1357	0.0607	0.0590	5,505,240
	(0.0441)	(0.0502)	(0.0197)	(0.0218)	
	[0.0000]	[0.0123]	[0.0043]	[0.0123]	

	Complete Blood Count		Total Protein		
Switch to:					
– Single	−0.1207	−0.2936	−0.0835	−0.1825	8,332,360
	(0.0728)	(0.1078)	(0.0400)	(0.0518)	
	[0.1223]	[0.0110]	[0.0569]	[0.0009]	
– Group	−0.1255	−0.3209	−0.0321	−0.1620	6,641,920
	(0.0773)	(0.1049)	(0.0531)	(0.0689)	
	[0.1423]	[0.0045]	[0.5802]	[0.0304]	
Switch to:					
– Larger	−0.0726	−0.3045	−0.0021	−0.1410	8,858,040
	(0.0685)	(0.0943)	(0.0443)	(0.0572)	
	[0.3390]	[0.0025]	[0.9616]	[0.0203]	
– Smaller	−0.1828	−0.3257	−0.1489	−0.2482	5,505,240
	(0.1036)	(0.1474)	(0.0540)	(0.0706)	
	[0.1147]	[0.0461]	[0.0118]	[0.0010]	

Notes: Standard Errors in Parentheses. Adjusted p-values following **?** with m = 34 (the number of presented point estimates per sub-sample), applied for each sub-sample separately, in brackets. *First Year* = Event Times 0 to 3, *Medium Term* = Event Times 4 to 12; *Switch to Single* = first practice a patient visits after the exit is a single practice. *Switch to Group* = first practice a patient visits after the exit is a group practice. *Switch to Larger* = first GP a patient visits after the exit has more patients than practice of leaving GP. *Switch to Smaller* = first GP a patient visits after the exit has less patients than practice of leaving GP.

Table S20: Effects by absorbing GP's practice characteristics – ambulatory care utilization II

	First Year	Med. run	First Year	Med. run	Observations
	Number of GP visit		Number of specialist visit		
Switch to:					
– Single	−0.0514	−0.0423	0.0460	0.0578	8,332,360
	(0.0087)	(0.0117)	(0.0077)	(0.0098)	
	[0.0000]	[0.0007]	[0.0000]	[0.0000]	
– Group	−0.0309	−0.0216	0.0562	0.0616	6,641,920
	(0.0094)	(0.0125)	(0.0089)	(0.0114)	
	[0.0023]	[0.1226]	[0.0000]	[0.0000]	
Switch to:					
– Larger	−0.0463	−0.0478	0.0454	0.0521	8,858,040
	(0.0082)	(0.0106)	(0.0074)	(0.0094)	
	[0.0000]	[0.0000]	[0.0000]	[0.0000]	
– Smaller	−0.0099	0.0157	0.0708	0.0868	5,505,240
	(0.0111)	(0.0159)	(0.0100)	(0.0126)	
	[0.4375]	[0.3893]	[0.0000]	[0.0000]	
	GP Costs		Specialist costs		
Switch to:					
– Single	−3.9107	0.0317	1.7558	2.5775	8,332,360
	(0.2933)	(0.3740)	(0.6290)	(0.8455)	
	[0.0000]	[0.9324]	[0.0094]	[0.0046]	
– Group	−1.6606	0.2608	2.5507	3.2392	6,641,920
	(0.3391)	(0.3968)	(0.7159)	(0.9298)	
	[0.0000]	[0.5605]	[0.0010]	[0.0012]	
Switch to:					
– Larger	−2.8220	0.1014	1.9319	2.7431	8,858,040
	(0.2864)	(0.3448)	(0.6192)	(0.8260)	
	[0.0000]	[0.7921]	[0.0033]	[0.0019]	
– Smaller	−2.5285	1.0037	3.3820	4.3141	5,505,240
	(0.3947)	(0.4907)	(0.8084)	(1.0702)	
	[0.0000]	[0.0661]	[0.0001]	[0.0001]	

Notes: Standard Errors in Parentheses. Adjusted p-values following **?** with m = 34 (the number of presented point estimates per sub-sample), applied for each sub-sample separately, in brackets. *First Year* = Event Times 0 to 3, *Medium Term* = Event Times 4 to 12; *Switch to Single* = first practice a patient visits after the exit is a single practice. *Switch to Group* = first practice a patient visits after the exit is a group practice. *Switch to Larger* = first GP a patient visits after the exit has more patients than practice of leaving GP. *Switch to Smaller* = first GP a patient visits after the exit has less patients than practice of leaving GP.

Table S21: Descriptive Statistics: Exit sample vs. late-exit sample

	Exit sample (used for main results) (exposed to a leaving GP)		Late-exit sample (exposed to a GP leaving in 2019Q4))		
	Mean	SD	Mean	SD	SMD
Patient characteristics:					
Birth Year	1958.026	15.641	1959.554	15.676	-0.069
Female	0.602	0.490	0.589	0.492	0.018
Rural	0.328	0.469	0.301	0.458	0.041
Age	51.974	15.641	50.446	15.676	0.069
Healthcare utilisation:					
Number of GP Visits	1.640	2.190	1.391	2.114	0.082
Any GP Visit	0.677	0.468	0.569	0.495	0.158
GP Costs [€]	44.500	66.230	38.094	53.120	0.076
Number of Specialist Visits	1.567	2.862	1.331	2.657	0.060
Any Specialist Visit	0.486	0.500	0.417	0.493	0.098
Specialist Costs [€]	64.763	204.927	55.520	186.947	0.033
Any Hospital Visit	0.028	0.165	0.022	0.148	0.026
Any Emergency Hospital Visit	0.014	0.116	0.011	0.105	0.017
Any Ambulatory Care Sensitive Condition	0.002	0.050	0.002	0.047	0.004
Diagnoses:					
Myocardial Infarction	0.008	0.088	0.006	0.081	0.004
Congestive Heart Failure	0.020	0.142	0.016	0.127	0.010
Peripheral Vascular Disease	0.028	0.165	0.021	0.144	0.016
Cerebrovascular Disease	0.029	0.167	0.024	0.152	0.012
Dementia	0.003	0.057	0.002	0.050	0.003
Chronic Pulmonary Disease	0.102	0.303	0.084	0.278	0.30
Rheumatoid Disease	0.019	0.137	0.015	0.121	0.012
Peptic Ulcer Disease	0.006	0.076	0.004	0.062	0.007
Mild Liver Disease	0.051	0.220	0.041	0.198	0.021
Diabetes Without Complications	0.065	0.247	0.050	0.218	0.030
Diabetes With Complications	0.017	0.130	0.014	0.115	0.010
Hemiplegia or Paraplegia	0.006	0.079	0.005	0.069	0.005
Renal Disease	0.015	0.120	0.011	0.106	0.009
Cancer (any Malignancy)	0.039	0.193	0.030	0.171	0.019
Moderate or Severe Liver Disease	0.001	0.025	0.0005	0.022	0.001
Cancer (metastatic solid tumour)	0.002	0.049	0.002	0.044	0.002
AIDS	0.001	0.024	0.001	0.024	-0.0001
Tests and Prescriptions:					
Any Blood Count	0.013	0.113	0.012	0.107	0.008
Any Total Protein	0.004	0.066	0.004	0.064	0.003
Any Beta Blocker	0.090	0.287	0.071	0.258	0.049
Any ACE Inhibitor	0.068	0.251	0.053	0.224	0.044
Any Antibiotics	0.013	0.112	0.011	0.103	0.012
Any Sick Note	0.148	0.356	0.134	0.341	0.029
Observations:	383,502		46,678		

Note: The estimation sample consists of individuals who are continuously insured and who all experience a GP exit between 2012 and 2017. The control sample consists of continuously insured individuals who experience a GP exit in the fourth quarter of 2019. Presented are observations from the first quarter in 2010. *Rural* = Individuals living in a county, where the share of inhabitants that live in municipalities with more than 150 Inhabitants per km^2 is less than 75% (?). Diagnoses indicate whether an individual has received the given diagnosis, based on the Charlson comorbidity index (?). Complete Blood Count as defined by EBM No.32122. Total Protein as defined by EBM No.32056. ACE Inhibitors include all prescriptions with ATC C09a and C09b. Beta Blockers include all prescriptions with ATC C07. Antibiotics include all prescriptions with ATC J01. SMD refers to the standardized mean differences and is calculated as follows: $SMD = \frac{Mean_1 - Mean_0}{\sqrt{SD_1 + SD_0}}$ (where the index 1 indicates the respective statistic for the estimation sample, while 0 refers to the control sample).
